# Fas apoptotic inhibitor molecule 2 mitigates metabolic dysfunction-associated fatty liver disease through autophagic CRTC2 degradation

**DOI:** 10.1038/s12276-025-01559-1

**Published:** 2025-10-07

**Authors:** Yongjie Yu, Sha Hu, Tuo Zhang, Hongjie Shi, Dajun Li, Yongping Huang, Yu Zhang, Haitao Wang, Yufeng Hu, Hong Yu, Guang-Nian Zhao, Peng Zhang

**Affiliations:** 1 Taikang Medical School (School of Basic Medical Sciences), Wuhan University, Wuhan, China; 2 Hubei Provincial Key Laboratory of Developmentally Originated Disease, Wuhan, China; 3 Department of Organ Transplantation, Renmin Hospital of Wuhan University, Wuhan, China; 4 Department of Cardiology, Zhongnan Hospital of Wuhan University, Wuhan, China; 5 State Key Laboratory of New Targets Discovery and Drug Development for Major Diseases, Gannan Innovation and Translational Medicine Research Institute, Gannan Medical University, Ganzhou, China; 6 Department of Obstetrics and Gynecology, National Clinical Research Center for Obstetrics and Gynecology, Tongji Hospital, Tongji Medical College, Huazhong University of Science and Technology, Wuhan, China

**Keywords:** Proteolysis, Metabolic disorders

## Abstract

Lysosomal membrane proteins play fundamental roles in the lysosomal degradation of proteins and are attractive drug targets for metabolic dysfunction-associated fatty liver disease (MAFLD). Fas apoptotic inhibitory molecule 2 (FAIM2), a lysosomal membrane protein, has been recognized as an inhibitor of apoptosis in a variety of diseases. Here we reveal that FAIM2 is an inhibitor of fatty acid synthesis and suppresses MAFLD. FAIM2 protein expression is decreased in MAFLD. Moreover, FAIM2 is degraded by the E3 ubiquitin ligase NEDD4L through the catalysis of K48-linked ubiquitination. High-fat and high-cholesterol diet-induced hepatic steatosis, inflammation and fibrosis are aggravated in Faim2-knockout mice and alleviated in mice with AAV8-mediated FAIM2 overexpression. Furthermore, in hepatocytes, FAIM2 knockout increases the expression of genes related to fatty acid synthesis, while overexpressing FAIM2 exhibits the opposite effect. Mechanistically, FAIM2 directly interacts with CREB-regulated transcription coactivator 2 (CRTC2), a prominent regulator of lipid metabolism, and mediates its degradation through autophagy. Specifically, we find that the N terminus of FAIM2, which interacts with CRTC2 and LC3, is required for autophagic degradation of CRTC2. Collectively, our findings reveal that FAIM2 acts as a fatty acid synthesis inhibitor in MAFLD by promoting the autophagic degradation of CRTC2 and that FAIM2–CRTC2 may be a promising therapeutic target.

## FAIM2 inhibits fatty acid synthesis in liver disease

Metabolic dysfunction-associated fatty liver disease (MAFLD) is a common liver condition affecting many people worldwide. Researchers are looking for new ways to treat MAFLD. A recent study explored the role of a protein called FAIM2 in MAFLD. They found that FAIM2 levels are lower in people with MAFLD, and this protein helps reduce fat buildup in the liver. Researchers used mice and primary hepatocytes to study the function of FAIM2. They discovered that, when FAIM2 is missing, fat and inflammation in the liver increase. However, increasing FAIM2 levels can reduce these issues. FAIM2 works by interacting with another protein, CRTC2, and helps break it down through a process called autophagy. The study suggests that boosting FAIM2 could be a new way to treat MAFLD by reducing fat buildup in the liver.

This summary was initially drafted using artificial intelligence, then revised and fact-checked by the author.

## Introduction

Metabolic dysfunction-associated fatty liver disease (MAFLD), previously known as nonalcoholic fatty liver disease, has become the most prevalent chronic liver disease, with a global prevalence rate of up to 30%, and its prevalence is increasing^1^. Moreover, MAFLD can progress from simple hepatic steatosis (simple fatty liver, MAFL) to metabolic dysfunction-associated steatohepatitis (MASH), which puts individuals at risk of end-stage liver diseases, such as cirrhosis and hepatocellular carcinoma^2^. Despite the large number of individuals with MAFLD, poor adherence to lifestyle interventions is observed in clinical practice^3^, and only limited pharmacological therapy has been approved for MASH^4^. Therefore, the identification of effective therapeutic targets and strategies for MAFLD is urgently needed.

Lysosomal degradation, including the endosome‒lysosome pathway and the autophagy‒lysosome pathway (also known as autophagy), is a pivotal physiological activity essential for maintaining cellular protein homeostasis and has been an appealing platform for drug discovery^5–7^. The membrane of lysosomes harbors a variety of proteins that play critical roles in maintaining lysosomal degradation (preserving the integrity of the lysosomal structure and regulating enzyme activity) and mediating interactions with other organelles or molecules^8–11^. The dysregulation of lysosomal membrane proteins can disrupt cellular protein homeostasis, which in turn can cause a spectrum of diseases, including MAFLD^12–14^. Ablation of glycosylated lysosomal membrane protein in mice promotes lipid deposition and causes fibrosis and hepatic cell death in the liver^15,16^. Moreover, the level of the lysosomal-associated protein transmembrane 5 is negatively correlated with the NAFLD activity score (NAS), and the hepatocyte-specific depletion of lysosomal-associated protein transmembrane 5 exacerbates MASH symptoms in mice^17^. Our previous studies revealed that transmembrane BAX inhibitor motif-containing protein 1 (TMBIM1) protects against MAFLD by promoting the lysosomal degradation of Toll-like receptor 4^18^. Given the crucial role of lysosomal membrane proteins in lysosomal degradation and their connections in MAFLD, lysosomal membrane proteins could be promising therapeutic targets for MAFLD treatment.

Fas apoptotic inhibitory molecule 2 (FAIM2), also known as TMBIM2, belongs to the TMBIM family, which is characterized by a UPF0005 motif that encodes either six or seven transmembrane domains. FAIM2 contains seven transmembrane domains and localizes to lysosomes^19^; it was initially identified in a study seeking genes involved in the development and maintenance of the nervous system^20^. Previous studies on FAIM2 have focused mainly on its antiapoptotic effect. Overexpression of FAIM2 protected cortical neurons from FasL-induced apoptosis and decreased caspase activation^21^, whereas knockdown of FAIM2 expression increased Fas-induced apoptotic cell death in small cell lung cancer cells^22^. In addition, FAIM2 protects hippocampal cells from death in the acute phase of bacterial meningitis^23^, reduces stroke volume and alleviates dopaminergic neuron degeneration in Parkinson’s disease^24,25^. Recently, multiple studies have suggested that single-nucleotide polymorphism mutations at the *FAIM2* gene locus are associated with obesity and type 2 diabetes, which are critical risk factors for MAFLD^26–29^. However, so far, the role of FAIM2 in MAFLD remains unknown.

Here, we identified FAIM2 as an effective suppressor of MAFLD. A marked decrease in FAIM2 protein levels was observed in patients with MAFLD. FAIM2 depletion exacerbated lipid deposition, inflammation and fibrosis in vivo. However, overexpressing FAIM2 in vivo had the opposite effect. Moreover, in hepatocytes, FAIM2 overexpression suppressed the expression of fatty acid synthesis- and inflammation-related genes. Mechanistically, FAIM2 directly interacted with CREB-regulated transcription coactivator 2 (CRTC2), a key regulator of lipid metabolism, and promoted the degradation of CRTC2 via the autophagy pathway. Collectively, our findings suggest that FAIM2 suppresses MAFLD by promoting the autophagic degradation of CRTC2 and further suppressing fatty acid synthesis, suggesting that FAIM2 could be utilized as a therapeutic target for MAFLD.

## Materials and methods

### Animal treatment

All animal protocols were approved by the Animal Care and Use Committee of Renmin Hospital of Wuhan University. The animals received humane care according to the criteria outlined in the Guide for the Care and Use of Laboratory Animals prepared by the National Academy of Sciences and published by the National Institutes of Health.

### Human liver samples

All procedures involving the collection of human samples have been approved by the Ethics Committee of Zhongnan Hospital of Wuhan University, and the principles of the Helsinki Declaration have been followed to obtain written informed consent from the participants or their family members. Human liver tissues were obtained from patients clinically undergoing liver biopsy, liver surgery or liver transplantation. Liver tissues excluding alcohol or viral hepatitis were subsequently subjected to hematoxylin and eosin (H&E) staining and scored by one to two expert pathologists.

### Statistical analysis

In brief, all statistical analyses were performed using SPSS 25.0 (IBM). For data showing a Gaussian distribution, parametric statistical analysis was performed using the two-tailed Student’s *t*-test for two groups. One-way analysis of variance (ANOVA) was applied to three or more groups, followed by either Bonferroni post-hoc analysis for data meeting homogeneity of variance requirements or Tamhane’s T2(M) post-hoc analysis for heteroscedastic data. For datasets with skewed distributions, nonparametric statistical analysis was performed using the Mann–Whitney *U* test for two groups and Kruskal–Wallis test for three or more groups. Data are presented as the mean ± s.d., and *P* < 0.05 was considered to indicate significance.

## Results

### FAIM2 expression is downregulated in fatty livers

**Fig. 1:**
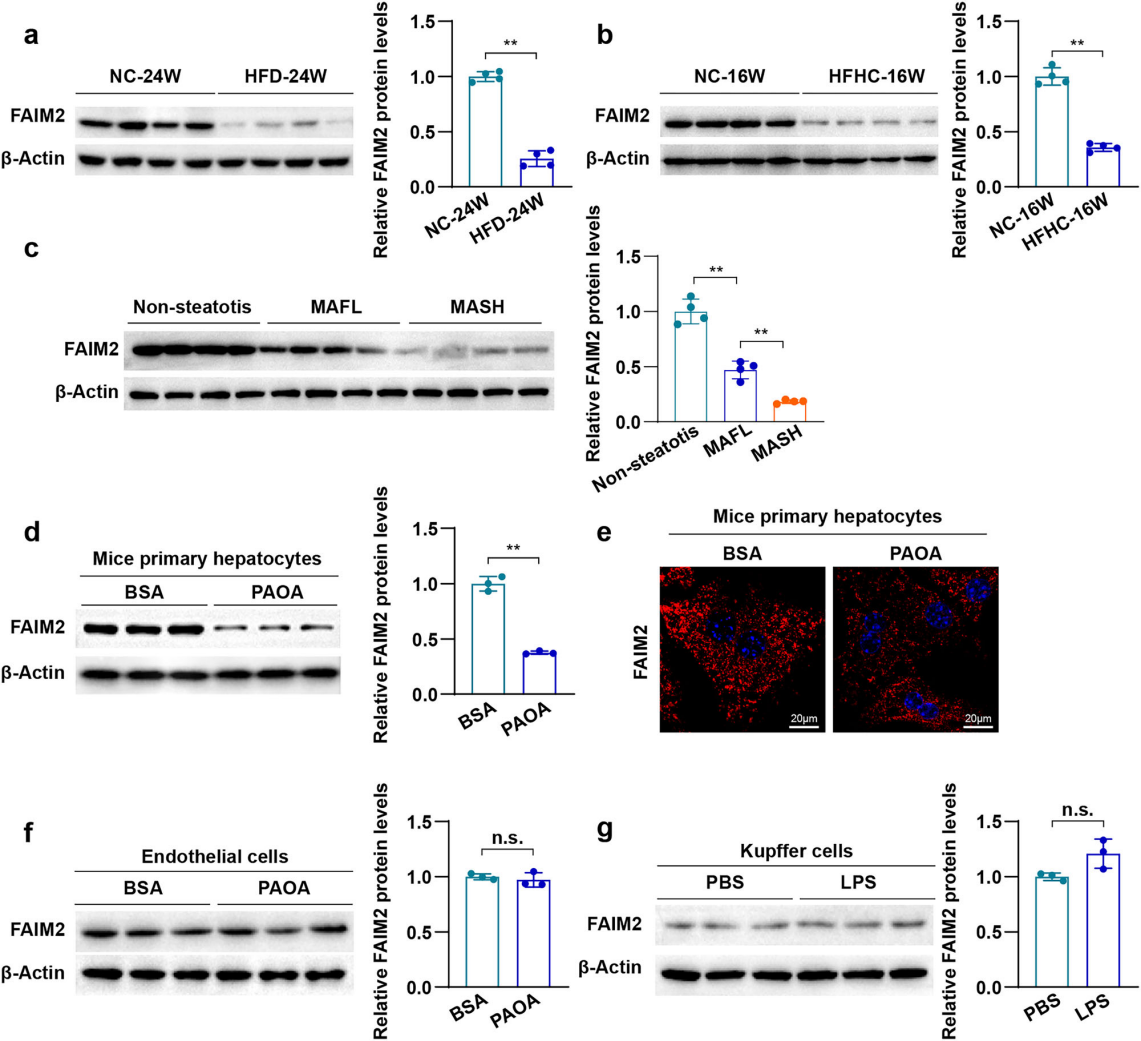
Correlations of FAIM2 expression with fatty liver diseases. **a**,**b** Western blot images and normalized quantification of FAIM2 expression in the livers of C57BL/6J mice that were fed a HFD or NC for 24 weeks (*n* = 4) (**a**) or mice that were fed a HFHC or NC for 16 weeks (*n* = 4) (**b**). **c** Western blot images and normalized quantification of FAIM2 expression from individuals with nonsteatotis, individuals with fatty liver (MAFL) and individuals with MASH (*n* = 4). **d** Western blot images and normalized quantification of FAIM2 expression in primary hepatocytes treated with palmitate and oil acid (PAOA, PA:OA = 0.5 mM:1 mM) (*n* = 3). **e** Images of FAIM2 immunofluorescence staining of primary hepatocytes treated with PBS or PAOA. Scale bars, 20 μm. **f**,**g** Western blot images and normalized quantification of FAIM2 expression in endothelial cells treated with PAOA (**f**) or Kupffer cells treated with LPS (**g**) (*n* = 3). **P < 0.01; n.s., not significant. The data were expressed as the means ± s.d. Statistical analysis was carried out via one-way ANOVA or two-tailed Student’s *t*-test.

To investigate whether FAIM2 was involved in MAFLD, we first examined FAIM2 expression in the liver. The results revealed that the FAIM2 protein levels were markedly lower in the livers of the mice fed a high-fat diet (HFD) for 24 weeks or a high-fat and high-cholesterol diet (HFHC) for 16 weeks than in those fed normal chow (NC) (Fig. 1a,b). Moreover, to further explore the relationship between FAIM2 and clinical MAFLD, we further examined the expression of FAIM2 in liver samples from individuals with MAFL or MASH (Supplementary Table 1). Consistent with the findings in mouse livers, compared with those in controls, the protein levels of FAIM2 were markedly lower in the livers of patients with MAFL and MASH **(**Fig. 1c). Considering that the occurrence and development of MAFLD were the result of complicated interactions among multiple cells in the liver, we further examined the expression of FAIM2 in the major cells of the liver. Surprisingly, compared with those in the respective controls, the protein levels of FAIM2 were markedly lower in palmitic acid- and oleic acid (PAOA)-stimulated hepatocytes, which were primarily in the cytoplasm (Fig. 1d,e), but FAIM2 protein levels were not significantly different in PAOA-treated endothelial cells or lipopolysaccharide (LPS)-stimulated Kupffer cells (Fig. 1f,g). In addition, the mRNA levels of FAIM2 were not significantly changed in the above MAFLD liver tissues or hepatocytes (Supplementary Fig. 1a). Taken together, the protein, but not the mRNA, levels of FAIM2 were significantly decreased in MAFLD livers and hepatocytes, suggesting that FAIM2 may be involved in the progression of MAFLD and regulated by posttranslational modifications during MAFLD.

### NEDD4L facilitates FAIM2 degradation through catalyzing its K48-linked ubiquitination

**Fig. 2:**
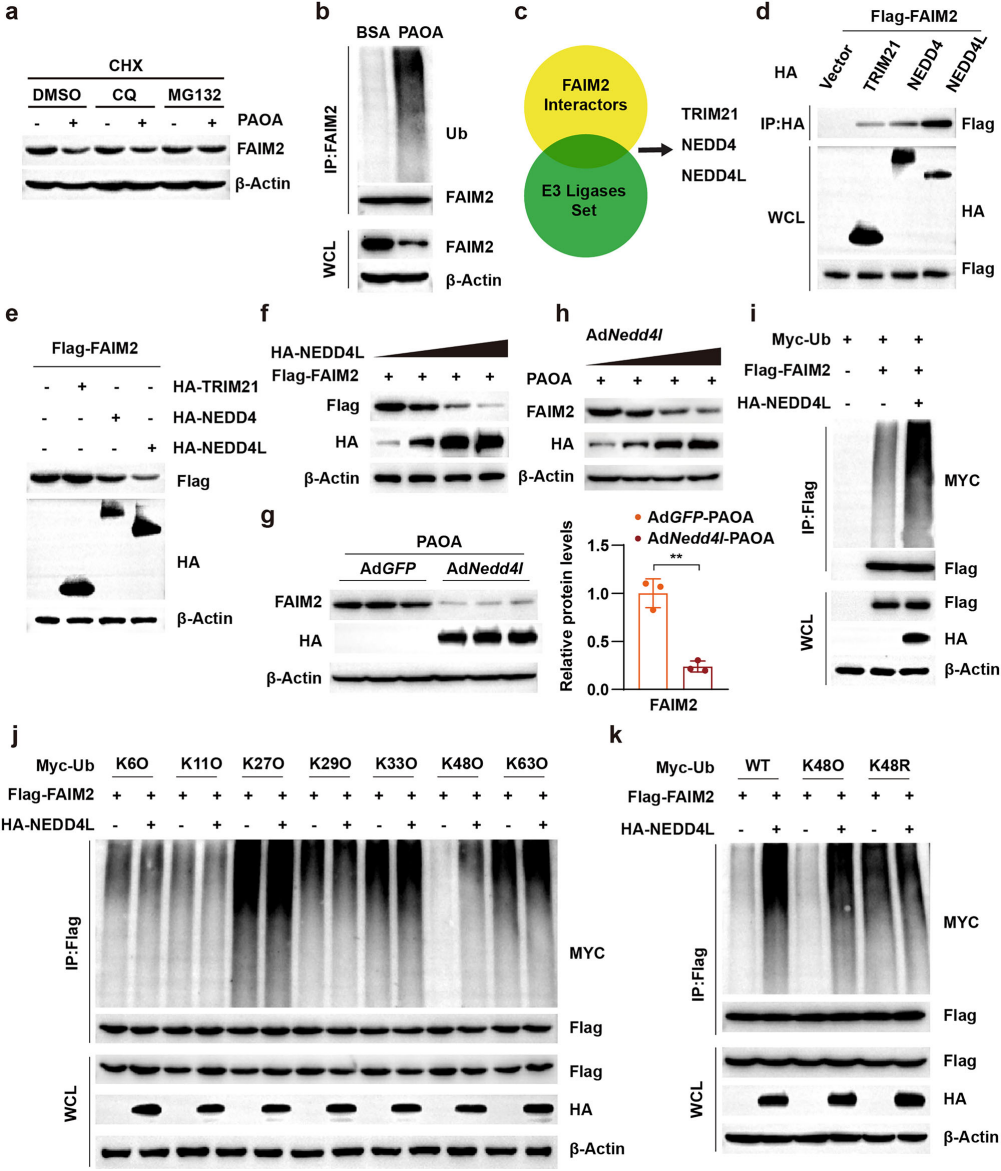
NEDD4L mediates FAIM2 degradation by catalyzing K48-linked ubiquitination. **a** Western blot images of FAIM2 expression in mouse hepatocytes treated with PAOA and CHX (50 μM) and MG132 (50 μM), CQ (50 μM) or dimethyl sulfoxide (DMSO). **b** Ubiquitination assays determining the ubiquitination of FAIM2 in primary hepatocytes. **c** Schematic illustration showing the process of screening E3 ligases that interact with FAIM2. **d** Interactions between FAIM2 and TRIM21, NEDD4 and NEDD4L in HEK293T cells. **e** Western blot images of FAIM2 expression in HEK293T cells transfected with the indicated plasmids for TRIM21, NEDD4L and NEDD4L. **f** Western blot images of FAIM2 expression in HEK293T cells transfected with increasing amounts of the NEDD4L plasmid. **g** Western blot images of FAIM2 expression in PAOA-treated and Ad*Nedd4l-*infected hepatocytes. **h** Western blot images of FAIM2 expression in the indicated hepatocytes infected with increasing amounts of the Ad*Nedd4l*. **i** Ubiquitination assays were used to determine the ubiquitination of FAIM2 after transfection with the indicated plasmids. **j** Screening of the ubiquitination of FAIM2 by NEDD4L with the indicated types of ubiquitin. **k** Ubiquitination of FAIM2 in HEK293T cells transfected with the indicated plasmids. **P < 0.01. The data are expressed as the means ± s.d. Statistical analysis was carried out via two-tailed Student’s *t*-tests.

To further elucidate the mechanism underlying the downregulation of FAIM2 protein levels in MAFLD, the mode of FAIM2 protein degradation was investigated. Because the ubiquitin‒proteasome pathway and lysosomal pathway were two major pathways responsible for intracellular protein degradation^30^, the proteasome pathway inhibitor MG132 and lysosomal pathway inhibitor chloroquine (CQ) were used to treat hepatocytes with PAOA, and the results revealed that the downregulation of FAIM2 was blocked by MG132 treatment (Fig. 2a). Moreover, the ubiquitination of FAIM2 was markedly increased under metabolic stress (Fig. 2b). The above results suggested that the downregulation of FAIM2 in MALFD was attributed to ubiquitin–proteasome-mediated FAIM2 degradation. E3 ligases are responsible for recognizing specific substrate proteins and committing them to proteasomal degradation^31^. To identify E3 ligases that might participate in the degradation of FAIM2, we intersected the FAIM2 interactors (BioGRID) and E3 ligase set, which revealed three candidates: TRIM21, NEDD4 and NEDD4L (Fig. 2c). Importantly, among these three E3 ligases, NEDD4L had the strongest interaction with FAIM2 (Fig. 2d) and mediated the most pronounced decrease in FAIM2 (Fig. 2e). Moreover, FAIM2 decreased with NEDD4L overexpression in a dose-dependent manner (Fig. 2f). Notably, in PAOA-induced hepatocytes, overexpression of NEDD4L also downregulated the protein level of FAIM2 in a dose-dependent manner (Fig. 2g,h). To further elucidate the mechanism of NEDD4L-mediated FAIM2 degradation, we performed ubiquitination assays. NEDD4L overexpression markedly promoted FAIM2 ubiquitination (Fig. 2i). Furthermore, experiments using mutant ubiquitin revealed that NEDD4L enhanced the K48-linked ubiquitination of FAIM2 (Fig. 2j,k). These results implied that the degradation of FAIM2 in hepatocytes under metabolic stress was attributed to NEDD4L-mediated K48-linked ubiquitination.

### FAIM2 KO aggravates HFD-induced metabolic disorders

**Fig. 3:**
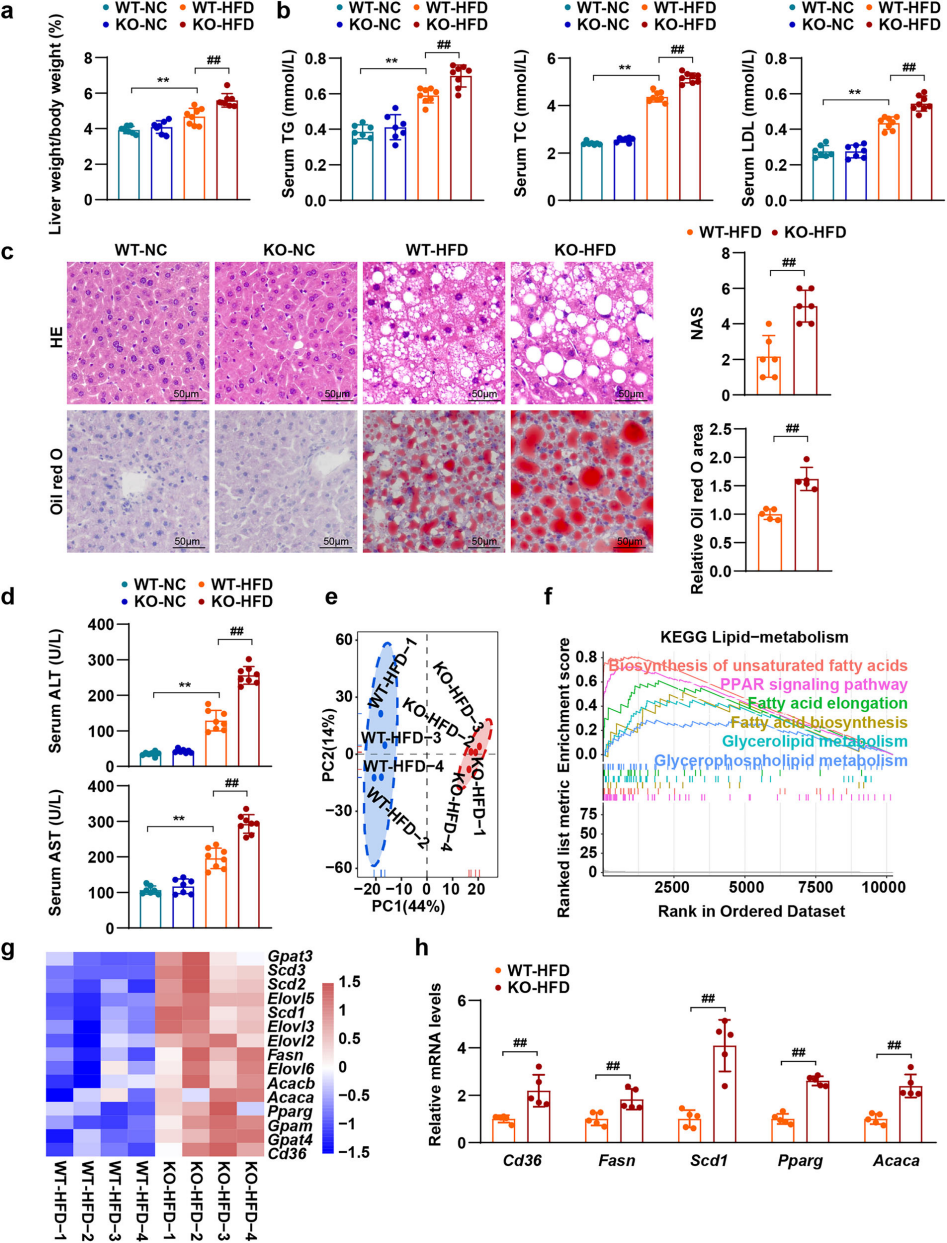
FAIM2 deletion exacerbates HFD-induced metabolic disorders. **a** Mouse liver weight/body weight percentages of NC-fed WT mice, NC-fed KO mice, HFD-fed WT mice and HFD-fed KO mice (NC group *n* = 7, HFD group *n* = 8). **b** Mouse absolute serum TG, TC and LDL levels in the indicated groups (NC group *n* = 7, HFD group *n* = 8). **c** Images and statistics of H&E-stained (top) and Oil Red O-stained (bottom) liver sections from the indicated groups (*n* = 5–6). Scale bars, 50 µm. **d** Serum absolute ALT and AST levels in the indicated groups (NC group *n* = 7, HFD group *n* = 8). **e**–**g** Analyses of PCA (**e**) GSEA (**f**) and heatmaps (**g**) of livers from Faim2-KO mice fed a HFD (GSEA pathways with *P* < 0.05; *n* = 4). **h** Normalized mRNA levels of genes related to fatty acid metabolism in the livers of the indicated groups. Gene expression was normalized to *Actb* mRNA levels (*n* = 5). **P < 0.01; ^##^*P* < 0.01. The data are expressed as the means ± s.d. Statistical analysis was carried out via one-way ANOVA or two-tailed Student’s *t*-test.

To explore the role of FAIM2 in the progression of MAFLD, Faim2-knockout (KO) mice and wild-type (WT) mice were fed a HFD or NC for 24 weeks (Supplementary Fig. 2a). The body weight of HFD-fed mice was greater than that of NC-fed mice, and the body weight of HFD-fed Faim2-KO (KO-HFD) mice was greater than that of WT mice after they were fed a HFD for 24 weeks (WT-HFD) (Supplementary Fig. 2b). Compared with WT mice, Faim2-KO mice fed a HFD for 24 weeks presented increased fasting blood glucose and more severe impaired glucose tolerance (Supplementary Fig. 2c–e). Moreover, FAIM2 deficiency further increased HFD-induced increases in liver weight (Supplementary Fig. 2f) and liver-to-body weight (Fig. 3a) as well as white adipose weight and white adipose-to-body weight (Supplementary Fig. 2g,h). Moreover, after HFD consumption for 24 weeks, the contents of serum triglyceride (TG), total cholesterol (TC) and low-density lipoprotein (LDL) in Faim2-KO mice were significantly greater than those in WT mice (Fig. 3b). The accumulation of hepatic lipids was markedly aggravated in Faim2-KO mice after HFD consumption, as indicated by H&E and Oil Red O staining (Fig. 3c). In addition, the higher serum alanine aminotransferase (ALT) and aspartate aminotransferase (AST) concentrations indicated exacerbated liver injury in the Faim2-KO mice than in the WT mice after HFD consumption (Fig. 3d).

RNA sequencing analyses were performed to further assess the influence of FAIM2 on MAFLD. Principal component analysis (PCA) clearly separated the HFD-treated liver samples into two subgroups (Fig. 3e). Notably, Gene Set Enrichment Analysis (GSEA) revealed that Kyoto Encyclopedia of Genes and Genomes (KEGG) pathways related to lipid metabolism, including fatty acid synthesis and elongation, were enriched in the KO-HFD group (Fig. 3f). Moreover, the genes related to these pathways were activated by FAIM2 deletion (Fig. 3g), which was confirmed by quantitative polymerase chain reaction (Fig. 3h). Taken together, these findings suggest that FAIM2 deficiency exacerbates HFD-induced metabolic disorders.

### FAIM2 KO aggravates HFHC-induced MASH

**Fig. 4:**
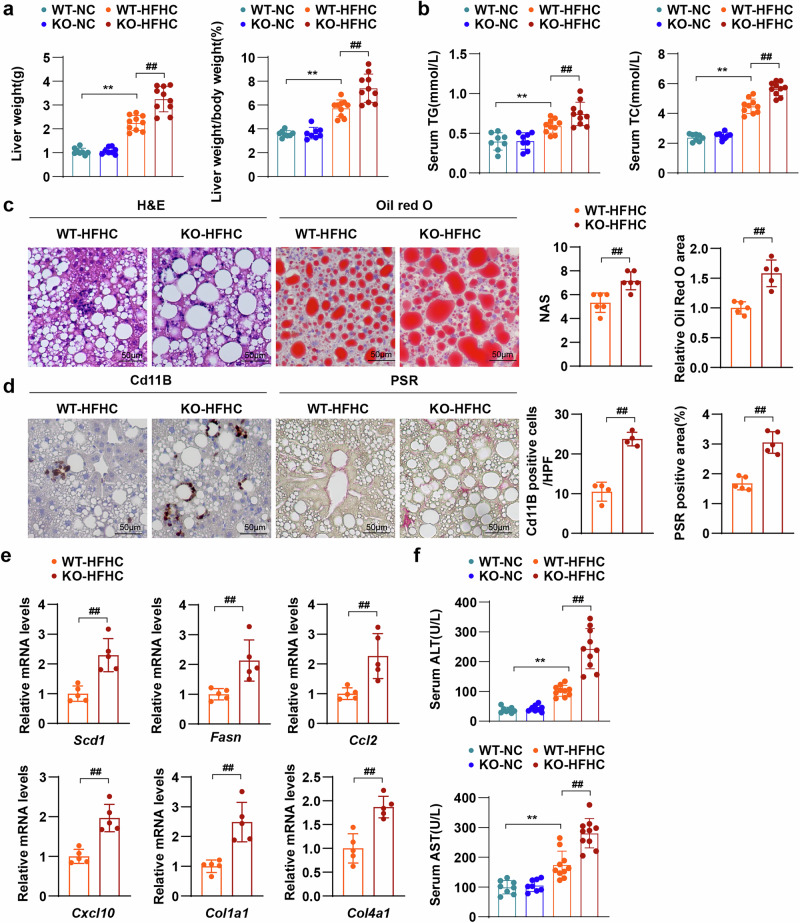
FAIM2 deletion exacerbates HFHC-induced MASH. **a** Mouse absolute liver weight and liver weight/body weight percentage of the indicated groups (NC group *n* = 8, HFHC group *n* = 10). **b** Mouse absolute serum TG and serum TC levels in the indicated groups (NC group *n* = 8, HFHC group *n* = 10). **c**,**d** Images of H&E, Oil Red O (**c**) Cd11B IHC and PSR liver sections (**d**) from the indicated groups (*n* = 4–6). Scale bars, 50 µm. HPF, high-power field. **e** Normalized mRNA levels of genes related to fatty acid synthesis (*Scd1* and *Fasn*), inflammation (*Ccl2* and *Cxcl10*) and fibrosis (*Col1a1* and *Col4a1*) in the livers of the indicated groups (*n* = 5). **f** Mouse absolute serum ALT and serum AST levels in the indicated groups (NC group *n* = 8, HFHC group *n* = 10). **P < 0.01; ^##^*P* < 0.01. The data are expressed as the means ± s.d. Statistical analysis was carried out via one-way ANOVA or two-tailed Student’s *t*-test.

MASH is a severe form of MAFLD that is characterized by hepatic steatosis, inflammation, ballooning and accompanied fibrosis and is more likely to progress to the end stage^32^. To investigate whether FAIM2 KO also accelerates the progression of MASH, Faim2-KO and WT mice were fed a HFHC diet for 16 weeks. Similar to HFD-fed mice, Faim2-KO mice presented greater body weights and blood glucose levels and more severe impaired glucose tolerance than WT mice did after being fed a HFHC diet (Supplementary Fig. 3a–c). After HFHC diet treatment for 16 weeks, Faim2-KO mice presented higher liver weights, liver-to-body weights (Fig. 4a), white adipose weights and white adipose-to-body weights than WT mice did (Supplementary Fig. 3d). In addition, higher serum lipid levels, such as TG and TC levels, as well as more severe hepatic steatosis in Faim2-KO mice than in WT mice fed a HFHC diet were also observed (Fig. 4b,c). Importantly, after 16 weeks of HFHC diet feeding, liver inflammation and collagen deposition were more severe in Faim2-KO mice than in WT mice (Fig. 4d). The mRNA levels of genes involved in lipid metabolism, inflammation and fibrosis were significantly increased in Faim2-KO mice under HFHC conditions (Fig. 4e). In addition, liver injury was exacerbated in Faim2-KO mice, as indicated by higher serum ALT and AST concentrations in Faim2-KO mice than in WT mice after they were fed a HFHC (Fig. 4f). Collectively, the above findings suggest that FAIM2 deficiency exacerbates HFHC-induced MASH.

### FAIM2 overexpression alleviates HFHC-induced MASH

To further evaluate whether FAIM2 overexpression also affects the process of MASH, FAIM2-overexpressing mice were generated by injecting adeno-associated virus 8 (AAV8) carrying the *Faim2* gene (AAV8-Faim2) into WT mice via the tail vein, and mice injected with control virus were used as controls (AAV8-Ctrl). Western blot analysis revealed that FAIM2 was highly expressed in liver tissues (Supplementary Fig. 4a). Compared with AAV8-Ctrl mice, AAV8-Faim2 mice fed a HFHC diet for 16 weeks presented lower body weights and blood glucose levels and improved glucose tolerance (Supplementary Fig. 4b–e). Notably, the liver weight, white adipose weight and ratio of these to body weight were lower in the AAV8-Faim2 mice than in the AAV8-Ctrl mice (Supplementary Fig. 4f–h). The contents of serum lipids, such as TG, TC and LDL, and the serum ALT and AST contents were also lower in the AAV8-Faim2 mice than in the AAV8-Ctrl mice (Supplementary Fig. 4i,j). Importantly, FAIM2 overexpression pronouncedly alleviated hepatic steatosis, inflammation and fibrosis in the AAV8-Faim2 mice (Supplementary Fig. 4k). Taken together, these findings suggest that FAIM2 overexpression mitigates HFHC-induced MASH.

### FAIM2 inhibits lipid deposition in hepatocytes

**Fig. 5:**
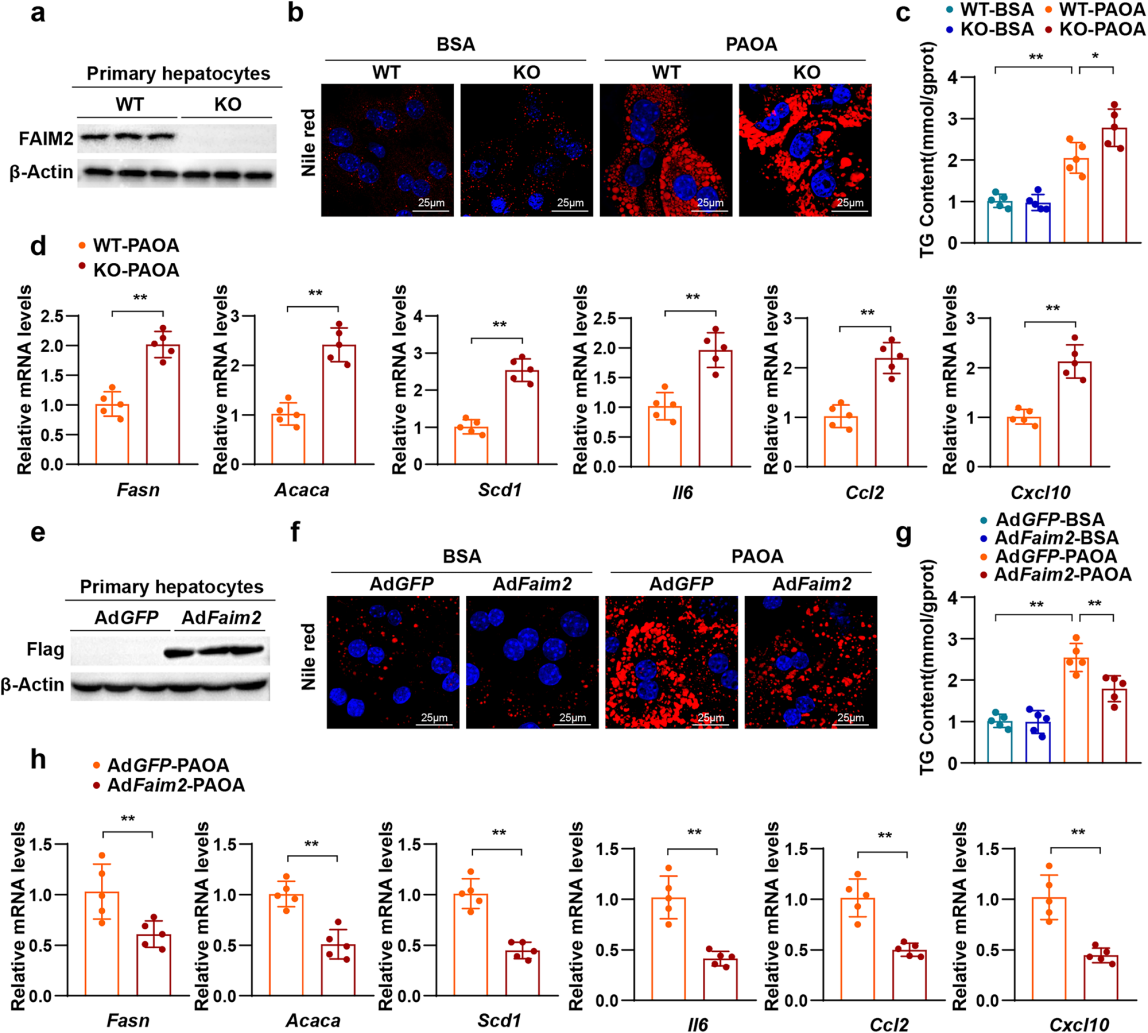
FAIM2 inhibits lipid deposition in hepatocytes. **a** Western blot images of FAIM2 protein expression in hepatocytes from FAIM2 KO and control mice (*n* = 3). **b** Nile red-stained images of hepatocytes from the indicated groups treated with PAOA or the bovine serum albumin (BSA) vehicle (*n* = 3). Scale bar, 25 μm. **c** Normalized TG content of hepatocytes treated with PAOA or the BSA vehicle in the indicated groups (*n* = 5). **d** Normalized mRNA levels of genes related to fatty acid synthesis and inflammation in primary hepatocytes from FAIM2-KO and control mice treated with PAOA (*n* = 5). **e** Western blot images of FAIM2 protein expression in hepatocytes infected with Ad*Faim2* or its control Ad*GFP* (*n* = 3). **f** Nile red-stained images of the indicated groups (*n* = 3). Scale bar, 25 μm. **g** Normalized TG content of the indicated groups (*n* = 5). **h** Normalized mRNA levels of the indicated groups (*n* = 5). **P < 0.01. The data are expressed as the means ± s.d. Statistical analysis was carried out via one-way ANOVA or two-tailed Student’s *t*-test.

Although the occurrence and development of MAFLD involve multiple cells in the liver, excessive lipid accumulation in hepatocytes is believed to be one of the major pathogeneses^33^. We observed that the protein levels of FAIM2 were downregulated in hepatocytes under metabolic stimulation, so we speculated that FAIM2 may affect the function of hepatocytes under metabolic stimulation. FAIM2-deficient primary hepatocytes were isolated from Faim2-KO mice, and western blot showed that FAIM2 expression was absent in primary hepatocytes (Fig. 5a). As indicated by Nile red staining and TG content tests, the lipid droplet and TG contents were markedly greater in FAIM2-deficient hepatocytes than in control hepatocytes under PAOA stimulation (Fig. 5b,c). Moreover, FAIM2 deletion promoted the expression of genes related to fatty acid synthesis and inflammation (Fig. 5d). Next, an adenovirus expressing FAIM2 (Ad*Faim2*) was constructed to investigate the function of FAIM2 overexpression in primary hepatocytes. Western blot showed that FAIM2 was successfully overexpressed in primary hepatocytes (Fig. 5e). By contrast, overexpression of FAIM2 reduced the lipid droplet and TG accumulation in hepatocytes induced by PAOA (Fig. 5f,g). The expression of genes related to fatty acid synthesis and inflammation was also inhibited by FAIM2 overexpression (Fig. 5h). Taken together, these findings indicate that FAIM2 inhibits lipid deposition and the inflammatory response in hepatocytes induced by PAOA.

### FAIM2 interacts with CRTC2 and reduces its expression

**Fig. 6:**
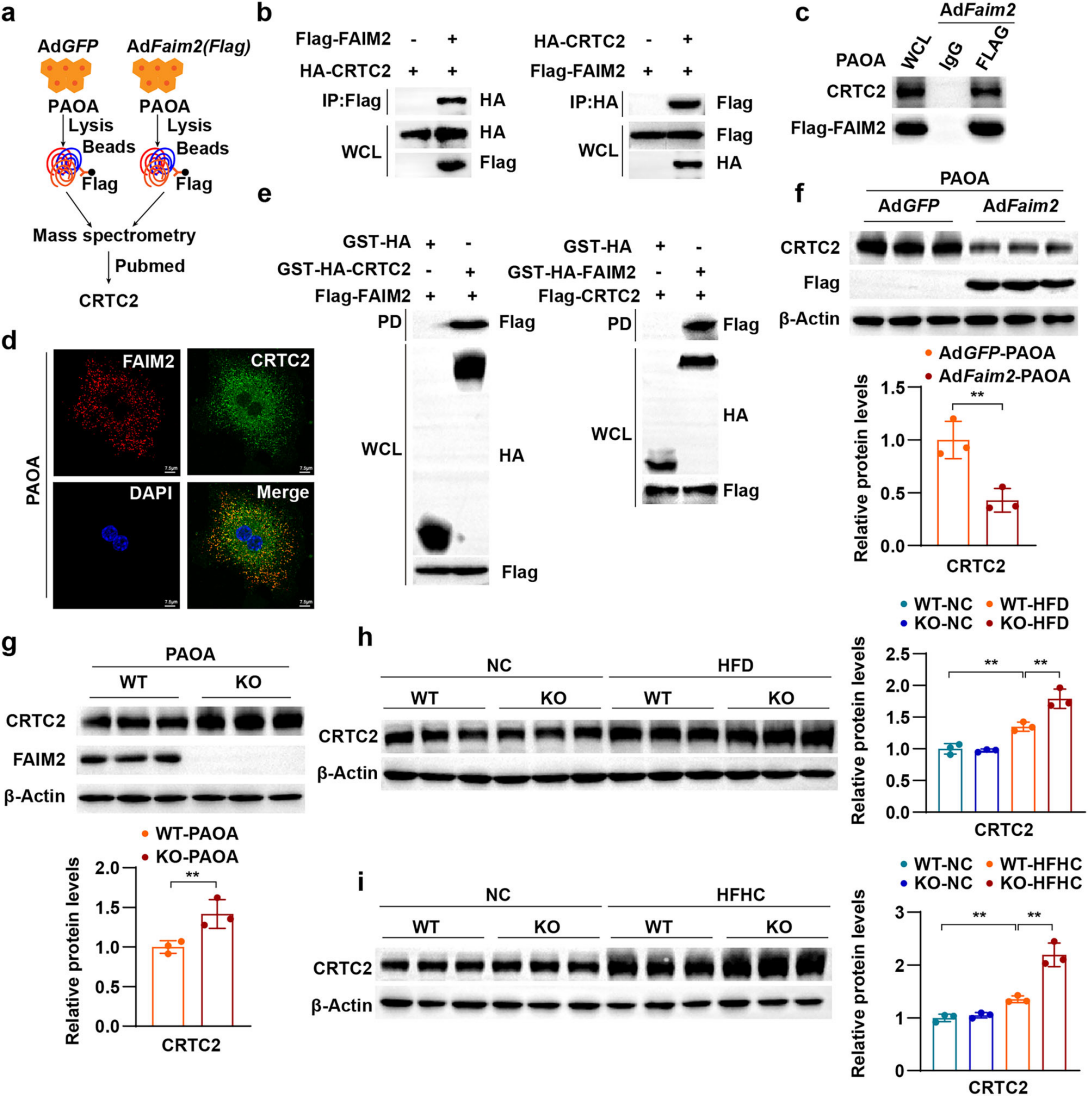
FAIM2 binds to CRTC2 and reduces its protein level. **a** Schematic diagram showing the IP‒MS analysis used to identify the specific target of FAIM2. **b**,**c** Immunoprecipitation and western blot analyses showing the binding of FAIM2 to CRTC2 in the indicated HEK293T cells (**b**) and PAOA-treated hepatocytes (**c**). **d** Confocal microscopy images showing colocalization between FAIM2 (red) and CRTC2 (green). Scale bar, 7.5 µm. **e** GST pulldown assay and western blot analyses showing the direct binding of FAIM2 to CRTC2 in HEK293T cells. **f** Normalized CRTC2 protein levels in isolatedWT hepatocytes infected with Ad*GFP* or Ad*Faim2* (*n* = 3). **g** Normalized CRTC2 protein levels in isolated WT hepatocytes and Faim2-KO hepatocytes (*n* = 3). **h** Normalized CRTC2 protein levels in the livers of NC- or HFD-fed Faim2-WT and Faim2-KO mice (*n* = 3). **i** Normalized CRTC2 protein levels in the indicated groups (*n* = 3). **P < 0.01. The data are expressed as the means ± s.d. Statistical analysis was carried out via one-way ANOVA or two-tailed Student’s *t*-test.

We next explored the mechanistic link between FAIM2 and its function. The above results revealed that FAIM2 KO substantially and consistently increased lipid accumulation and upregulated fatty acid synthesis-related gene expression in in vivo and in vitro MAFLD models, whereas FAIM2 overexpression had the opposite effects. Thus, we speculated that FAIM2 may function by affecting lipid metabolism regulators. We applied immunoprecipitation-mass spectrometry (IP‒MS) to identify proteins that physically interact with FAIM2 and screened out lipid metabolism regulators. Among them, CRTC2 has been reported to be a key lipid metabolism regulator that promotes MAFLD^34–36^ (Fig. 6a). The interaction and colocalization of FAIM2 and CRTC2 in PAOA-stimulated hepatocytes were verified (Fig. 6b–d). The results of the GST pulldown assay indicated a direct interaction between FAIM2 and CRTC2 (Fig. 6e). Although the mRNA levels of CRTC2 in PAOA-induced hepatocytes were not affected by the overexpression or loss of FAIM2 (Supplementary Fig. 5a,b), its protein levels were significantly decreased by FAIM2 overexpression and increased by FAIM2 deficiency (Fig. 6f,g). Consistent with the in vitro results, CRTC2 mRNA levels were comparable, while CRTC2 protein levels were significantly greater in the livers of HFD- or HFHC-fed Faim2-KO mice than in those of WT mice (Fig. 6h,i and Supplementary Fig. 5c,d). Moreover, CRTC2 expression was increased in the livers of individuals with MAFLD (Supplementary Fig. 5e,f). Collectively, these findings suggest that FAIM2 directly interacts with CRTC2 and decreases its protein level, indicating that FAIM2 may regulate lipid metabolism via CRTC2 in hepatocytes.

### FAIM2 regulates lipid metabolism via CRTC2

To evaluate whether CRTC2 is required for FAIM2-regulated lipid metabolism in hepatocytes, we constructed an adenovirus overexpressing CRTC2 (Ad*Crtc2*). Hepatocytes were then infected with Ad*Faim2*, Ad*Crtc2* or both viruses together (Supplementary Fig. 6a). The inhibitory effect of FAIM2 overexpression on lipid accumulation was blunted by CRTC2 overexpression (Supplementary Fig. 6b,c). Overexpressing CRTC2 also reversed the inhibitory effects of FAIM2 on the expression of genes related to fatty acid synthesis and inflammation (Supplementary Fig. 6d). Next, we constructed adenovirus with short hairpin RNA targeting CRTC2 (Adsh*Crtc2*) and infected hepatocytes isolated from Faim2-KO mice and WT mice (Supplementary Fig. 6e). Similarly, CRTC2 knockdown reversed not only the aggravated lipid accumulation but also the activation of genes related to lipid metabolism and inflammation in hepatocytes induced by FAIM2 KO under PAOA treatment (Supplementary Fig. 6f–h). These data indicate that the inhibitory effect of FAIM2 on lipid metabolism and inflammation in hepatocytes requires CRTC2.

### FAIM2 facilitates CRTC2 degradation through autophagy

**Fig. 7:**
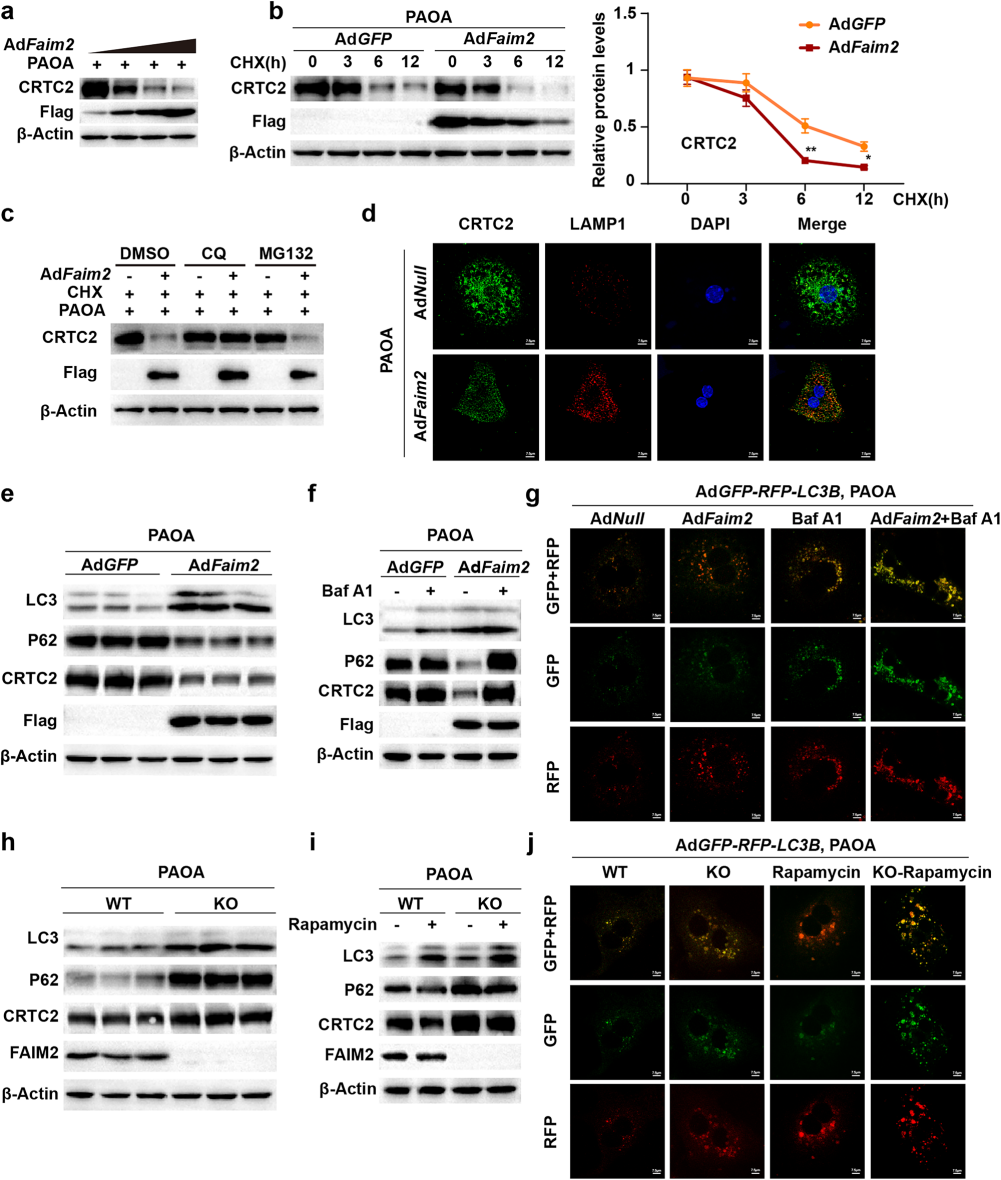
FAIM2 promotes CRTC2 degradation via autophagy. **a** Western blot images of CRTC2 and Flag-FAIM2 in primary hepatocytes infected with Ad*Faim2* and treated with PAOA. **b** Western blot images and normalized quantification of the indicated groups treated with PAOA and CHX (50 μM). **c** Western blot images of the indicated groups treated with DMSO, MG132 (50 μM) or CQ (50 μM) for 6 h. **d** Confocal microscopy images showing the distribution of CRTC2 (green) and LAMP1 (red) in the indicated hepatocytes with PAOA treatment. Scale bar, 7.5 µm. **e** Western blot images of LC3, P62, CRTC2 and Flag-FAIM2 in WT hepatocytes infected with Ad*GFP* or AdFaim2 under PAOA treatment. **f** Western blot images of LC3, P62, CRTC2 and Flag-FAIM2 in the indicated WT hepatocytes treated with or without Baf A1 (100 nM). **g** Images of primary hepatocytes infected with Ad*GFP-RFP-LC3B* in the indicated groups. Scale bar, 7.5 µm. **h** Western blot images of LC3, P62, CRTC2 and FAIM2 in PAOA-treated WT or Faim2-KO hepatocytes. **i** Western blot images of LC3, P62, CRTC2 and FAIM2 in the indicated groups treated with or without rapamycin (10 µM). **j** Images of the indicated groups infected with Ad*GFP-RFP-LC3B*. Scale bar, 7.5 µm. *P < 0.05; **P < 0.01. The data are expressed as the means ± s.d. Statistical analysis was carried out via one-way ANOVA.

We next sought to determine how FAIM2 decreases the protein level of CRTC2. The protein level of CRTC2 decreased with increasing FAIM2 expression in a dose-dependent manner (Fig. 7a). Notably, CRTC2 was degraded faster in FAIM2-overexpressing hepatocytes than in control hepatocytes under PAOA and cycloheximide (CHX) treatment (Fig. 7b). In addition, CQ treatment blocked the degradation effect of FAIM2 on CRTC2, and FAIM2 overexpression promoted the colocalization of CRTC2 with the lysosomal marker lysosome-associated membrane glycoprotein 1 (LAMP1) (Fig. 7c,d). These results suggest that FAIM2 promotes the degradation of CRTC2 via the lysosomal pathway. The lysosomal pathway includes the endosome‒lysosome pathway and autophagy^37^. Autophagy, in particular, is principally responsible for the degradation of cytoplasmic components^38,39^. Considering that FAIM2 and CRTC2 were localized mainly in the cytoplasm, we hypothesized that FAIM2 might facilitate CRTC2 degradation through autophagy. FAIM2 overexpression markedly promoted the degradation of CRTC2 and enhanced autophagic flux in hepatocytes treated with PAOA, as indicated by LC3 upregulation and P62 downregulation (Fig. 7e). In addition, FAIM2 overexpression substantially promoted autophagy, as indicated by the further accumulation of LC3 and p62 in hepatocytes infected with Ad*Faim2* under bafilomycin A1 (Baf A1) stimulation and the increased number of red fluorescent markers representing autolysosomes in PAOA-induced hepatocytes infected with Ad*Faim2* (Fig. 7f,g). More importantly, Baf A1 reversed the degradation of CRTC2 caused by FAIM2 overexpression (Fig. 7f). Conversely, the deletion of FAIM2 increased the protein level of CRTC2 and disrupted the autophagic flux in hepatocytes induced by PAOA (Fig. 7h). Moreover, Faim2-KO hepatocytes showed more intense yellow fluorescence of GFP–RFP–LC3B (reflecting autophagosome accumulation) and higher LC3 expression upon treatment with the autophagic flux activator rapamycin (Fig. 7i,j), suggesting that FAIM2 is required for autolysosome formation in hepatocytes. Taken together, these results suggest that FAIM2 promotes autophagy in hepatocytes and mediates CRTC2 degradation through autophagy.

### The N-terminal domain of FAIM2 is required for CRTC2 degradation

**Fig. 8:**
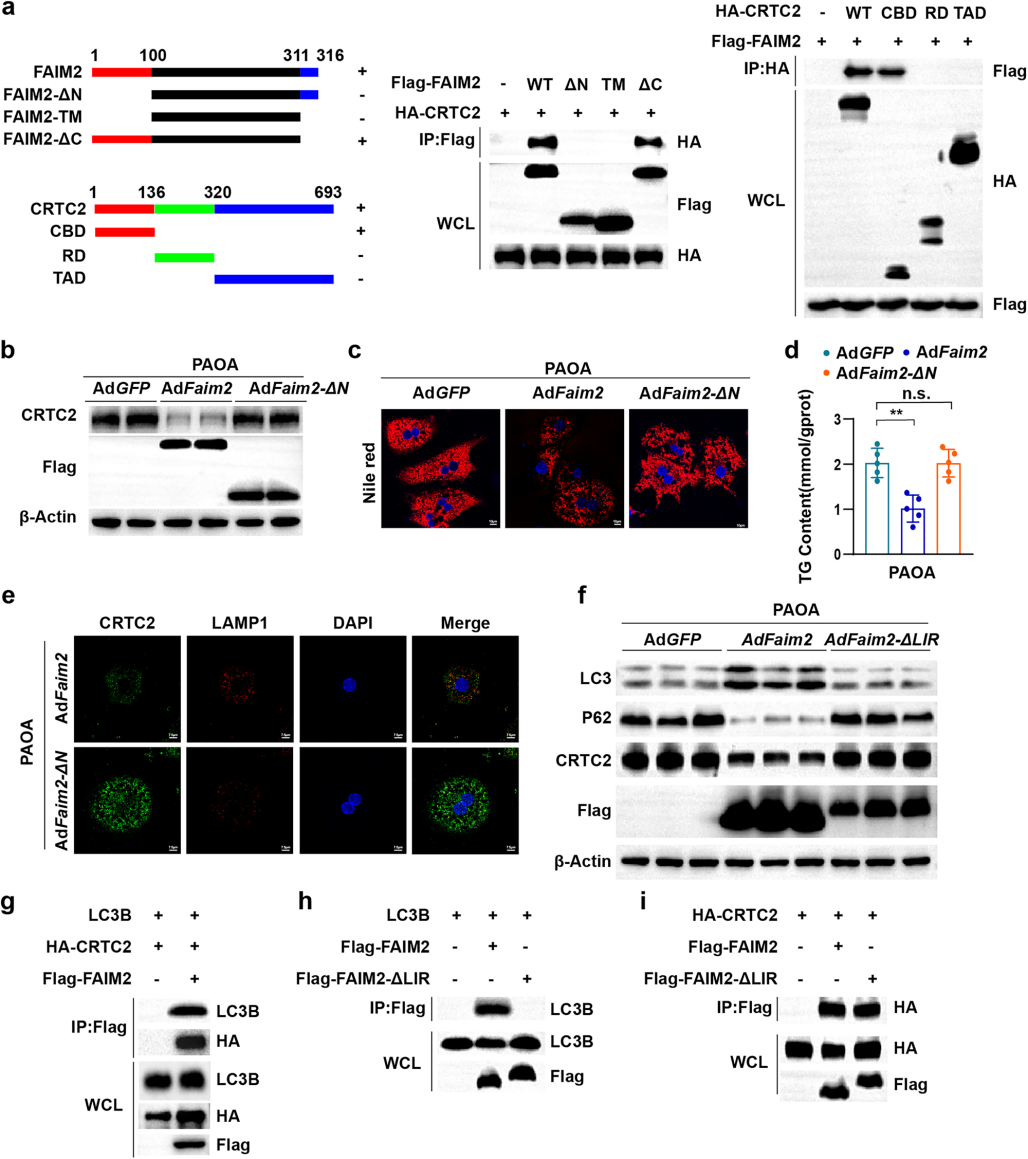
The N-terminal domain of FAIM2 is required for CRTC2 degradation. **a** Schematic illustration showing full-length and truncated FAIM2 and CRTC2 with Co-IP assays for mapping analyses of the domains responsible for the FAIM2–CRTC2 interaction. ΔN, missing N-terminal domain; TM, transmembrane domain; ΔC, missing C-terminal domain; CBD, CREB-binding domain; RD, regulation domain; TAD, transactivation domain. **b** Western blot images of CRTC2 in WT hepatocytes infected with Ad*GFP*, Ad*Faim2* or Ad*Faim2-ΔN*. **c** Nile red-stained images of hepatocytes from the indicated groups (*n* = 3). Scale bar, 10 µm. **d** Absolute TG content levels of the indicated groups (*n* = 5). **e** Confocal microscopy images of primary hepatocytes infected with Ad*Faim2* or Ad*Faim2-ΔN*. Scale bar, 7.5 µm. **f** Western blot images of the indicated groups. **g** Immunoprecipitation and western blot analyses showing the binding relationships between LC3B, CRTC2 and FAIM2B in HEK293T cells transfected with plasmids encoding LC3B, HA-CRTC2, and Flag-FAIM2. **h** Immunoprecipitation and western blot analyses showing the binding relationships between FAIM2, FAIM2-ΔLIR and LC3B in HEK293T cells transfected with the indicated plasmids. **i** Immunoprecipitation and western blot analyses showing the binding relationships between CRTC2, FAIM2 and FAIM2-ΔLIR in HEK293T cells transfected with the indicated plasmid. **P < 0.01; n.s., not significant. The data are expressed as the means ± s.d. Statistical analysis was carried out via one-way ANOVA.

To further investigate the detailed mechanism by which FAIM2 mediates CRTC2 degradation, the interaction domain between FAIM2 and CRTC2 was investigated. The results of domain co-Immunoprecipitation (Co-IP) analysis revealed that the N-terminal domain of FAIM2 interacted with the CREB binding domain (CBD) of CRTC2 (Fig. 8a). To determine whether the interaction between FAIM2 and CRTC2 is necessary for the protective role of FAIM2 against lipid metabolism, an adenovirus containing FAIM2 lacking the N-terminal domain (Ad*Faim2-ΔN*) was constructed. Interestingly, the deletion of the N-terminal domain of FAIM2 impaired its function in reducing CRTC2 levels (Fig. 8b), inhibiting lipid accumulation (Fig. 8c,d) and downregulating fatty acid synthesis- and inflammation-related genes in PAOA-treated hepatocytes. (Supplementary Fig. 7a). In addition, FAIM2-ΔN failed to promote CRTC2-lysosome colocalization or respond to CQ (Fig. 8e and Supplementary Fig. 7b). These results indicate that the inhibitory effects of FAIM2 on CRTC2 and metabolic stress require the N-terminal domain of FAIM2.

The N-terminal domain of FAIM2 contains an LC3-interacting region (LIR) motif that is responsible for autolysosome formation^19^. Therefore, we further investigated whether the interaction between FAIM2 and CRTC2 is dependent on the LIR motif. We constructed a plasmid and an adenovirus to overexpress FAIM2 lacking the LIR domain (FAIM2-ΔLIR). FAIM2-ΔLIR lost its ability to promote autophagy and suppress CRTC2 in hepatocytes induced by PAOA (Fig. 8f). FAIM2 was able to interact with both CRTC2 and LC3B, whereas FAIM2-ΔLIR was unable to bind with LC3B (Fig. 8g,h). However, FAIM2-ΔLIR was still able to interact with CRTC2 (Fig. 8i), indicating that the interaction between FAIM2 and CRTC2 was not dependent on the LIR motif. Taken together, these results suggest that the N-terminal domain of FAIM2 interacts with both CRTC2 and LC3, a process that is required for the suppression of CRTC2 by FAIM2.

## Discussion

In the present study, we revealed that the FAIM2 protein level was downregulated in MAFLD. In addition, FAIM2 was degraded through the ubiquitin‒proteasome pathway under metabolic stress. The E3 ligase NEDD4L enhanced the K48-linked ubiquitination of FAIM2 and promoted its degradation. Moreover, the protein levels of NEDD4L have been reported to be upregulated in human individuals with MASH and mouse MASH models^17^. Thus, the underlying mechanism of reduced FAIM2 expression may be that NEDD4L expression is elevated in the liver by metabolic stimuli such as a HFD or HFHC diet. This increase allows more NEDD4L to interact with FAIM2 and promote the K48-linked ubiquitination and ubiquitin‒proteasome degradation of FAIM2, leading to a decreased protein level of FAIM2 in MAFLD.

To elucidate the function of FAIM2 in MAFLD, we subjected Faim2-KO mice, AAV8-Faim2 mice and isolated Faim2-KO and Faim2-overexpressing hepatocytes to MAFLD models. FAIM2 deficiency markedly increased lipid deposition and upregulated pathways and genes related to fatty acid synthesis. However, the overexpression of FAIM2 had the opposite effect. These findings suggest that FAIM2 plays a protective role in MAFLD by mitigating fatty acid synthesis in hepatocytes and highlight the important role of lysosomal membrane proteins in MAFLD and other metabolism-related diseases.

We speculated that FAIM2 may mitigate fatty acid synthesis by affecting lipid metabolism regulators. Through immunoprecipitation‒mass spectrometry, we identified lipid metabolism regulators that interacted with FAIM2. Among these regulators, CRTC2 is a crucial lipid metabolism regulator in the liver. Liver-specific KO of CRTC2 reduces fatty acid synthesis gene expression (*Fasn*, *Scd1*, *Pparg* and *Acaca*), alleviates diet-induced liver lipid accumulation, inhibits inflammation^34^ and leads to reduced blood lipid and blood glucose concentrations^35^. In addition, CRTC2 overexpression enhances liver cholesterol synthesis by promoting the transcription of SREBP-2^36^. CRTC2 regulates fatty acid, glucose and cholesterol metabolism, making it a nonnegligible target. We verified that FAIM2 facilitated CRTC2 degradation through autophagy and that the N-terminal domain of FAIM2 was required for this process, which elucidated the underlying mechanism of FAIM2 in MAFLD and provided a potential therapeutic target for further clinical translation.

We propose that targeting CRTC2 for degradation is the primary cellular mechanism underlying the function of FAIM2 in MAFLD. However, CRTC2 regulates not only fatty acid biosynthesis^34^, but also blood glucose levels and cholesterol synthesis^36,40^, and participates in the processes of type 2 diabetes, stroke, tumors and circadian rhythm^41–44^. Indeed, the complete disruption of an important target that has such fundamental roles in physiological activity might lead to serious side effects, thus greatly hindering the development of new therapeutic agents. Our results demonstrated that FAIM2 facilitated the autophagic degradation of CRTC2, thereby preventing its excessive activation under prosteatotic stimulus, which works by balancing CRTC2 dynamics to maintain CRTC2 expression at an appropriate level rather than severely blocking its function. This working mode would probably be beneficial for treating MAFLD without inducing serious side effects. Moreover, we demonstrate that FAIM2-mediated autophagic degradation of CRTC2 alleviates hepatocyte lipid deposition, potentially inhibiting lipotoxicity-induced apoptosis—a mechanism that may importantly supplement FAIM2’s known antiapoptotic functions

Earlier work demonstrated that FAIM2 promotes autophagy by binding to LC3^19^. Notably, in addition to inhibiting lipid synthesis, research has revealed an increase in lipophagy, which can be attributed at least partly to the alleviation of fatty liver symptoms by liver-specific CRTC2 KO. Mechanistically, HFD feeding activated the CRTC2‒mTORC1 axis and impaired autophagic flux in the liver, whereas liver-specific KO of CRTC2 inhibited this process^34^. Thus, FAIM2-mediated autophagic degradation of CRTC2 may weaken the CRTC2‒mTORC1 axis, which in turn amplifies the pro-autophagic function of FAIM2. However, this hypothesis requires further investigation.

Our previous study revealed that TMBIM1 is an effective inhibitor of MAFLD. TMBIM1 collaborates with the endosomal sorting complex required for transport (ESCRT) machinery to promote multivesicular body formation, thereby facilitating the lysosomal degradation of TLR4 and exerting its protective function^18^. Although both TMBIM1 and FAIM2 belong to the TMBIM family, we found that FAIM2 protects against MAFLD by mediating the autophagic degradation of CRTC2. The different underlying mechanisms of TMBIM1 and FAIM2 indicate that they do not simply serve as functional substitutes.

Although we have used various models and experimental approaches to elucidate the function and mechanism of FAIM2 in MAFLD, there remains room for refinement in our study. We have demonstrated that FAIM2 can act through primary hepatocytes. However, owing to the limitations of global KO, we cannot exclude the potential effects of extrahepatic contributions. Liver-specific KO of Faim2 is warranted to further strengthen our conclusion. Specifically, Faim2 KO in Kupffer cells and infiltrating myeloid cells may affect the inflammatory phenotype. Future studies using cell-specific Faim2-KO models are needed to clarify their respective roles in inflammation. We observed that HFD-KO mice exhibited fasting hyperglycemia and impaired glucose tolerance. Further analyses, such as serum insulin measurements or insulin tolerance tests, would help to distinguish between insulin resistance and β-cell dysfunction. Moreover, we observed increased body weight in KO-HFD mice, and altered feeding behavior may contribute to weight gain, potentially confounding the phenotype. As food intake and energy expenditure were not recorded, we cannot currently determine whether the increased body weight of KO-HFD mice results from altered feeding behavior or from adipocytes Faim2 KO. Future studies incorporating rigorous dietary management and energy expenditure measurements, as well as adipocyte-specific Faim2-KO mice, are warranted to elucidate this issue. In addition, the use of a molecular docking assay to predict and validate the specific amino acid sequence within the N terminus of FAIM2 where CRTC2 binds and the use of artificial intelligence technology to design small-molecule compounds for therapeutic testing may pave the way for clinical translation.

In summary, this study reveals the role and mechanism of FAIM2 in MAFLD. Under metabolic stress, FAIM2 mediates the degradation of CRTC2 via autophagy and further suppresses fatty acid synthesis and alleviates MAFLD. Therefore, FAIM2–CRTC2 may hold promise as a novel therapeutic target for MAFLD, and targeting lysosomal membrane proteins to control protein homeostasis may provide new strategies and promising therapeutic targets.

## Supplementary information

Supplementary Information

Supplementary Fig. 1

Supplementary Fig. 2

Supplementary Fig. 3

Supplementary Fig. 4

Supplementary Fig. 5

Supplementary Fig. 6

Supplementary Fig. 7

## Data Availability

The data that support the findings of this study are available from the corresponding author upon reasonable request.
