# Supplementary material for: Fas apoptotic inhibitor molecule 2 mitigates metabolic dysfunction-associated fatty liver disease through autophagic CRTC2 degradation
Source: Exp Mol Med. 2025 Oct 7;57(10):2331–43. doi: 10.1038/s12276-025-01559-1 (PMC12586438; doi:10.1038/s12276-025-01559-1)
Supplement: Supplementary file 1 — Supplementary Information [file 12276_2025_1559_MOESM1_ESM.pdf]

Fas apoptotic inhibitor molecule 2 mitigates metabolic dysfunction-associated fatty liver disease through autophagic CRTC2 degradation

Yongjie Yu[1,2], Sha Hu[1,3*], Tuo Zhang[1,2*], Hongjie Shi[1,4], Dajun Li[1,2], Yongping Huang[1,2], Yu Zhang[1,2], Haitao Wang[1,2], Yufeng Hu[5], Hong Yu[1,2#], Guang-Nian Zhao[6#], Peng Zhang[1,2#]*

[1]Taikang Medical School (School of Basic Medical Sciences), Wuhan University, Wuhan 430071, China.

[2]Hubei Provincial Key Laboratory of Developmentally Originated Disease, Wuhan 430071, China.

[3]Department of Organ Transplantation, Renmin Hospital of Wuhan University, Wuhan 430060, China.

[4]Department of Cardiology, Zhongnan Hospital of Wuhan University, Wuhan 430071, China.

[5]State Key Laboratory of New Targets Discovery and Drug Development for Major Diseases, Gannan Innovation and Translational Medicine Research Institute, Gannan Medical University, Ganzhou 341000, China.

[6]Department of Obstetrics and Gynecology, National Clinical Research Center for Obstetrics and Gynecology, Tongji Hospital, Tongji Medical College, Huazhong University of Science and Technology, Wuhan430030, China.

[*]These authors contributed equally to this work.

[#]Correspondence authors:

Name: Peng Zhang; **Address:** Taikang Medical School (School of Basic Medical Sciences), Wuhan University, 115 Donghu Road, Wuchang District, Wuhan 430071, Hubei Province, China; **E-mail:**

zhp@whu.edu.cn.

Name: Guang-Nian Zhao; **Address:** National Clinical Research Center for Obstetrics and Gynecology, Tongji Medical College of HUST, Affiliated Tongji Hospital, 1095Jiefang Avenue, Qiaokou District, Wuhan 430030, Hubei Province, China; **E-mail:** zhaogn@tjh.tjmu.edu.cn.

Name: Hong Yu; **Address:** Taikang Medical School (School of Basic Medical Sciences), Wuhan University, 115 Donghu Road, Wuchang District, Wuhan 430071, Hubei Province, China; **E-mail:** yu.hong@whu.edu.cn.

Supplementary materials and methods

Animal models. Faim2-KO mice (Strain NO. T017435) were purchased from GemPharmatech, China. 8- to 10-week-old male mice (20-25 g) were maintained in a standard environment with a 12-h light–dark cycle. The fatty liver model was established in mice by feeding them a high-fat diet (protein, 20%; fat, 45%; carbohydrates, 35%; Medicience, MD12032, China) for 24 weeks or high-fat and high-cholesterol diet (protein, 14%; fat, 42%; carbohydrates, 44%; cholesterol, 2%; Trophic, IMA2019001, China) for 16 weeks. Mice that were administered a normal chow diet (protein, 21%; fat, 12%; carbohydrates, 67%; Xietong Shengwu, XT101WC-004, China) served as controls. To overexpress FAIM2, the 2×10^{11}vg adeno-associated Virus 8 (AAV8) in 200 μL phosphate buffered saline (PBS) was injected via tail vein to wild-type mice, with empty vector as control.

Human liver grouping and exclusion criteria. Simple steatosis and MASH were diagnosed using standard histological criteria by one to two pathologists, as follows: samples with a NAS of 1–2, ballooning scores of 0 and no fibrosis were classified as those with simple steatosis; samples with a NAS ≥ 5 or a NAS of 3–4 but with fibrosis were included in the MASH group; samples with a NAS of 0 were classified as nonsteatotic. Patients with liver steatosis resulting from excessive alcohol consumption, from drug/toxin use or viral infection (e.g., hepatitis B (HBV) and C virus (HCV) infections) were excluded from the study. Demographic data and histological scores of each human liver sample are provided in Supplementary Table1.

Tolerance tests. We carried out glucose tolerance test on the mice that had been fasted for 6 h. After determining the fasting blood glucose levels, we intraperitoneally injected each animal with glucose (1 g glucose/kg body weight; Baxter, A6E0152, USA). Blood glucose levels were measured after 15, 30, 60,

and 120 min.

Serum lipid and liver enzyme assay. The lipid content including triglyceride, total cholesterol, low-density lipoprotein cholesterol, and liver function, evaluated based on the alanine transaminase (ALT) and aspartate aminotransferase (AST) levels in the serum, were examined using a Hitachi automatic biochemical analyzer (Hitachi, Hitachi3110, Japan) according to the manufacturer's instructions.

Immunohistochemistry. Immunohistochemistry analyses were performed using paraffin-embedded sections of tissue samples. After antigen retrieval processing, the mouse liver tissue sections were washed with a 3% hydrogen peroxide solution at room temperature for 20 min. Subsequently, the sections were incubated with 10% bovine serum albumin at 37℃ for 30 min. Then, the primary antibody CD11B (Boster, BM3925, diluted 1:4000, China) was added to the sections and incubated overnight at 4℃. Following this, the sections were rinsed with PBS and treated with a goat anti-mouse detection kit (Biolight, BLRE006-200T, China) at 37℃ for 1 h. After washing with PBS, positive signals on the sections were observed using a diaminobenzidine working solution (ZSGB-BIO, ZLI-9018, China). The sections were then stained with hematoxylin (Servicebio, G1004, China) and washed with deionized water. Finally, the sections were sealed with resin mounting medium (Baso, BA-7004, China), and images were captured using an ordinary light microscope (Nikon, ECLIPSE 80i, Japan).

Histological analyses. Liver sections were embedded in paraffin and then stained with H&E to visualize the pattern of lipid accumulation and the inflammatory status. Lipid droplet accumulation was visualized using Oil Red O (Sigma-Aldrich, O0625, USA) staining of frozen liver sections that were prepared in Tissue-Tek OCT compound. Liver fibrosis was assessed via picrosirius red (PSR; Hede Biotechnology Co., Ltd., 26357-02, China) staining. The histological features of the tissues were observed and imaged

using a light microscope (Nikon, ECLIPSE 80i, Japan).

Primary cell isolation and adenovirus infection. Wild-type or Faim2-knockout mice underwent anesthesia before exposing their livers. After the tissues were perfused and digested by liver perfusion medium (Thermo Fisher Scientific, 17701-038, USA) and liver digestion medium (Thermo Fisher Scientific, 17701-034, USA) respectively, digestion was stopped when diffuse hepatic parenchyma was observed. The liver was then excised, minced, and filtered through a 70 μm cell strainer (Falcon, 352350, USA). The filtrate was placed in a centrifuge and spun at 50 g for 5 min. The supernatant was removed and the cells were resuspended in complete medium. The number of viable cells was then counted, and finally, the cells were plated. At the same time, primary Kupffer cells and sinus endothelial cells were obtained from the supernatant by gradient centrifugation. All Cells were cultured at 37℃ in an incubator containing 5% CO_2. In subsequent experiments, the primary hepatocytes were infected with appropriate adenovirus 6 h.

Cell lines. HEK293T and HEK293A cells were purchased from the Type Culture Collection of the Chinese Academy of Sciences, Shanghai, China. Mycoplasma contamination was checked, and the results were negative.

Plasmid constructs. Plasmids encoding human FAIM2 were obtained by cloning the cDNA encoding FAIM2 into the pHAGE-Flag, pcDNA5-Flag and pcDNA5-GST-HA vectors, respectively. The sequences encoding mutant FAIM2 (aa1-311, aa101-311, aa101-316, ΔLIR) were cloned into the pcDNA5 vector. The full-length region of human CRTC2 was cloned into pHAGE-HA, pHAGE-Flag or pcDNA5-GST-HA vector separately. The plasmids encoding mutant CRTC2 (aa1-144, aa136-320, aa316-693) were constructed by cloning the indicated coding regions into the pcDNA5-HA vector. The

primers used for plasmid construction are listed in Supplementary Table 2.

Adenoviral vector construction. To overexpress mouse FAIM2, the entire coding region of the mouse FAIM2 gene was placed into a replication-defective adenoviral vector under the control of the cytomegalovirus promoter. The entire coding region of the mouse NEDD4L and CRTC2 was placed into a replication-defective adenoviral vector too. To knock down mouse CRTC2 expression, three mouse CRTC2-specific short hairpin RNA (shRNA)-squenes were used to construct Adsh*Crtc2* adenoviruses. Ad*GFP* or AdshRNA were used as controls. Recombinant adenoviruses were generated using the EZRecombinase™ LR Enzyme Mix (GeneCopoeia, ER001, USA). Plasmids were recombined with the pAd/PL-DEST backbone vector, according to the manufacturer's instructions. After digestion with PacI enzyme (BBI, B600754-0025, Canada), the plasmids were transfected into HEK293A cells using FuGENE transfection reagent (Roche, E2312, Switzerland). Recombinant adenoviruses were plaque-purified, titered to 109 plaque-forming units per mL. The primers used for adenoviral vector construction are listed in Supplementary Table 2. AdPlus-*mCherry*-*GFP-LC3B* (Ad*GFP-RFP-LC3B*) was purchased from Beyotime (Beyotime, C3012-1Ml, China). Ad*Faim2-ΔN* was purchased from Designgene (Designgene, DGVD57, China).

Adeno-associated virus 8 (AAV8) construction. AAV8-Faim2 was purchased from the Designgene (Designgene, DG8A728, China). The construction method was followed: to construct the adeno-associated virus 8 of Faim2, the entire coding region of the mouse Faim2 gene was cloned into destination vector pAAV-TBG-3flag-IRES-ZsGreen. Using the three-plasmid AAV packaging system—consisting of the constructed pAAV-TBG-mFaim2-3flag-IRES-ZsGreen plasmid, serotype plasmid pRC8, and helper plasmid pHelper—we transfected the plasmids into HEK293-AAV cells to

produce high-titer viruses. The viruses were purified by ultracentrifugation, and their titers were quantified via qPCR.

qPCR. For qPCR assays, total RNA was extracted from mouse liver tissue or primary hepatocytes using TRIzol reagent (Invitrogen, 15596-026, USA). The mRNA was then reverse transcribed into cDNA using HiScript III RT SuperMix (Vazyme, R323-01, China). Following this, the expression of target genes was quantified by using ChamQ SYBR qPCR Master Mix (Vazyme, Q311-02, China) and the LightCycler 480 II instrument (Roche, Switzerland) under standard conditions. *Actb* was used as the reference gene, and the primer sequences for the target genes are listed in Supplementary Table 3.

RNA-Seq and data processing. For the RNA-Seq assay, cDNA libraries were constructed, and single-end libraries were sequenced by MD2000. HISAT2 software (version 2.21, USA) was used to align clean reads to Ensembl mouse (mm10 or GRCm38) genomes. Then, SAMtools (version 1.4, China) was stored and used to convert the aligned reads to Binary Alignment Map (BAM) format. Fragments per kilobase per million mapped fragments (FPKM) and read counts of each identified gene were calculated by StringTie (version 1.3.3b, USA).

Western blot analysis. The samples of liver tissue and cells were lysed using an SDS lysis buffer containing protease inhibitors (50 mM Tris-HCl pH 6.8, 2% SDS, 10% glycerol, 0.1 M DTT) to obtain total protein. Protein concentration was then determined using a BCA Protein Assay Kit (Thermo Fisher Scientific, 23225, USA). Subsequently, SDS-PAGE electrophoresis was performed to separate equal amounts of protein within the gel. The separated proteins were transferred onto a PVDF membrane (Millipore, IPVH00010, USA), which was then blocked for 1 h with a solution of 5% skimmed milk in TBST (Tris-buffered saline and 0.1% Tween-20). The PVDF membrane was incubated overnight at 4℃

with the corresponding primary antibody, followed by incubation for 1 h at room temperature with the appropriate secondary antibody. Finally, the PVDF membrane containing the target protein was incubated with an enhanced chemiluminescence reagent (Bio-Rad, 170-5061, China), and detection was carried out using a ChemiDoc MP Imaging System (Bio-Rad, USA). Protein grayscale quantification was performed using ImageJ software. β-Actin was used as a control, and detailed information on all used antibodies can be found in Supplementary Table 4.

Immunofluorescence staining. To study protein interactions, primary hepatocytes were incubated with FAIM2 (Santa Cruz Biotechnology, sc-398737, USA) or CRTC2 (Proteintech, 12497-1-AP, China), LAMP1(Cell Signaling Technology, 15665, USA). In brief, after 12 h of PAOA (PA:OA = 0.5:1 mM) stimulation, primary hepatocytes were fixed for 30 min with 4% paraformaldehyde solution (Servicebio, G1101-500ML, China). Subsequently, the hepatocytes were washed with PBS and then permeabilized with 0.2% Triton X-100 (Sigma-Aldrich, T8787, USA) for 5 min. They were then blocked at 37℃ with 8% goat serum (Abbkine, BMS0050, China). Next, the hepatocytes on slides were incubated with the corresponding antibodies, followed by treatment with fluorescent secondary antibodies of the appropriate species. Finally, images were acquired using a confocal fluorescence microscope (Leica, LCS-SP8-STED, Germany).

Nile red staining. Primary hepatocytes were stimulated with PAOA and then washed with PBS solution. Subsequently, they were fixed at room temperature for 30 min with a 4% formaldehyde solution (Servicebio, G1101-500ML, China). The cells were then incubated with Nile red solution (Beyotime, Y261772-100mg, China) at room temperature for 10 min, followed by three washes with PBS. Afterward, the cells were mounted with DAPI (Beyotime, P0131-5mL, China) and imaged using a confocal

fluorescence microscope (Leica, LCS-SP8-STED, Germany).

Triglyceride detection. The triglyceride assay was performed using a Triglyceride Assay Kit (Nanjing Jiancheng, A110-1-1, China) according to the manufacturer's protocol. After treatment, the cells were lysed with 1% Triton X-100 solution (Beyotime, P0096-100mL, China). A portion of the lysate was mixed with the assay working solution, and the absorbance was measured. Another portion of the lysate was centrifuged, and the supernatant was collected to determine protein concentration. The triglyceride content was calculated using the formula provided by the manufacturer.

Immunoprecipitation (IP) assay. After co-transfection of HEK293T cells with indicated plasmids and corresponding adenoviruses infected primary hepatocytes, cells were treated with cold IP lysis buffer (20 mM Tris HCl, pH 7.4; 150 mM NaCl; 1 mM EDTA; and 1% Triton X-100) containing protease inhibitor (Roche, 04693132001, Switzerland) and phosphatase inhibitor (Roche, 4906837001, Switzerland). Samples were centrifuged at 4℃ for 10 min and a small part of supernatant was boiled with 4 × SDS lysis buffer as whole cell lysis (WCL), and the rest supernatant containing protein was incubated with protein G Bestarose 4FF beads (Bestchrom, AA104307, China) and indicated labeled antibody overnight at 4℃. Finally, the beads were washed with cold IP buffer containing 150 mM or 300 mM NaCl for three times respectively. Then the beads were boiled in 2 × SDS loading buffer at 95℃ for 10 min prior to western blotting analysis. To conduct IP-mass spectrometry analysis, the precipitated proteins were separated by SDS-PAGE gels and then delivered to Shanghai Bioprofile Technology for detection.

Glutathione-S-transferase (GST) precipitation assays. Lysis of HEK293T cells transfected with indicated plasmids was obtained with lysis buffer (50 mM Na_2HPO_4, 300 mM NaCl, 1% Triton) containing protease inhibitor cocktail tablets (Roche, 04693132001, Switzerland) and purified by

Glutathione Sepharose 4B beads (GE Healthcare, 45-000-139, China), Flag antibodies and Flag peptide (Sigma-Aldrich, F4799, USA). Purified GST-HA tagged protein was incubated with Glutathione Sepharose 4B beads for 3 h at 4℃ and washed by cold GST buffer (20 mM Tris-HCl pH 6.8, 150 mM NaCl, 0.2% TritonX-100) for three times, before being mixed with purified Flag tagged protein for overnight at 4℃. The beads were washed by cold GST buffer for three times and western blotting analysis was conducted after precipitated proteins boiled in 2 × SDS loading buffer at 95℃ for 10 min.

LC3B fluorescence assays. Primary hepatocytes were infected with Ad*GFP-RFP-LC3B* and stimulated with PAOA, followed by washing with PBS solution. They were then fixed at room temperature for 30 min with a 4% formaldehyde solution (Servicebio, G1101-500ML, China). After another PBS wash, the cells were mounted with glycerin solution and imaged using confocal microscopy (Leica, LCS-SP8-STED, Germany) to observe fluorescence changes.

Supplementary figures

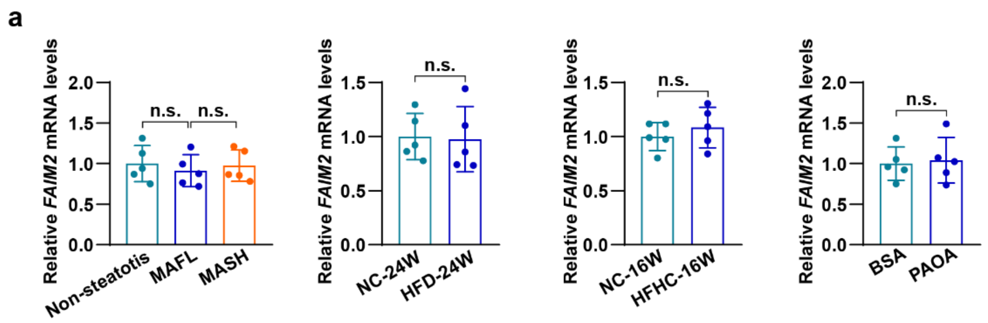

Supplementary Fig. 1: Correlations of FAIM2 expression with fatty liver diseases.

(a) qPCR analyses of the normalized mRNA levels of FAIM2 in fatty livers of human, HFD-fed mice, HFHC-fed mice and primary hepatocytes of wild-type mice (n = 5). n.s., not significant. The data were expressed as the means ± standard deviations. Statistical analysis was carried out via one-way ANOVA or two-tailed Student's t test.

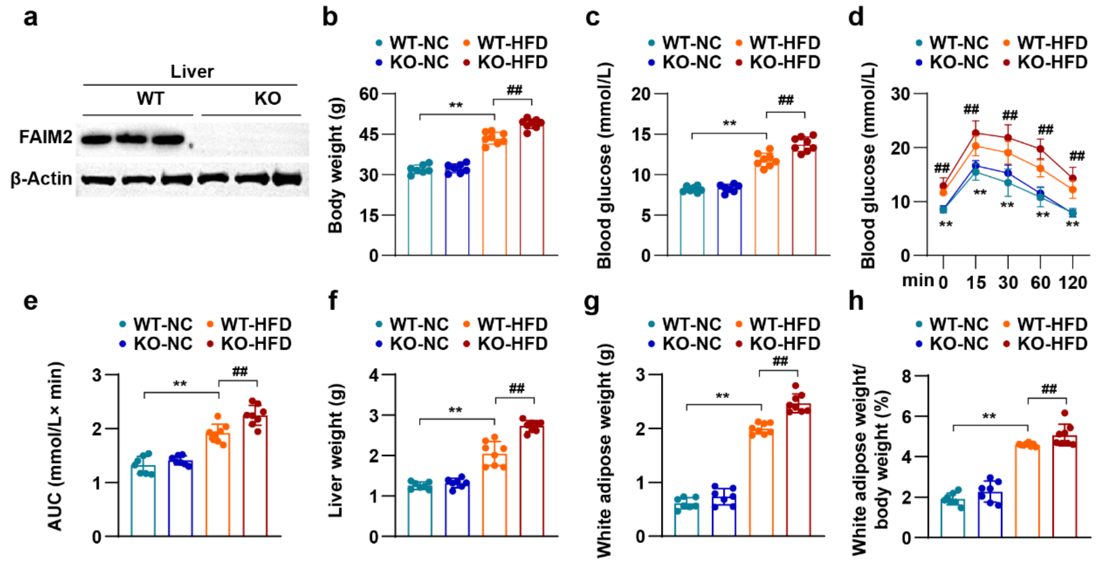

Supplementary Fig. 2: FAIM2 deletion exacerbates HFD-induced metabolic disorders.

(a) Western blot images of FAIM2 protein expression in liver from FAIM2 knockout and control mice (n = 3). **(b)** Absolute body weight of FAIM2-WT and FAIM2-KO mice that were fed NC or HFD (n = 7/NC

group, n=8/HFD group). **(c-e)** Mouse absolute (c) blood glucose levels, (d) blood glucose levels in a glucose tolerance test (GTT) and (e) GTT area under curve (AUC) of the indicated groups (n = 7/NC group, n=8/HFD group). **(f-h)** Mouse (f) absolute liver weight, (g) absolute white adipose weight, (h)white adipose/ body weight percentage of the indicated groups (n = 7/NC group, n=8/HFD group). $^{**}p < 0.01$; $^{\#\#}p < 0.01$. The data were expressed as the means ± standard deviations. Statistical analysis was carried out via one-way ANOVA.

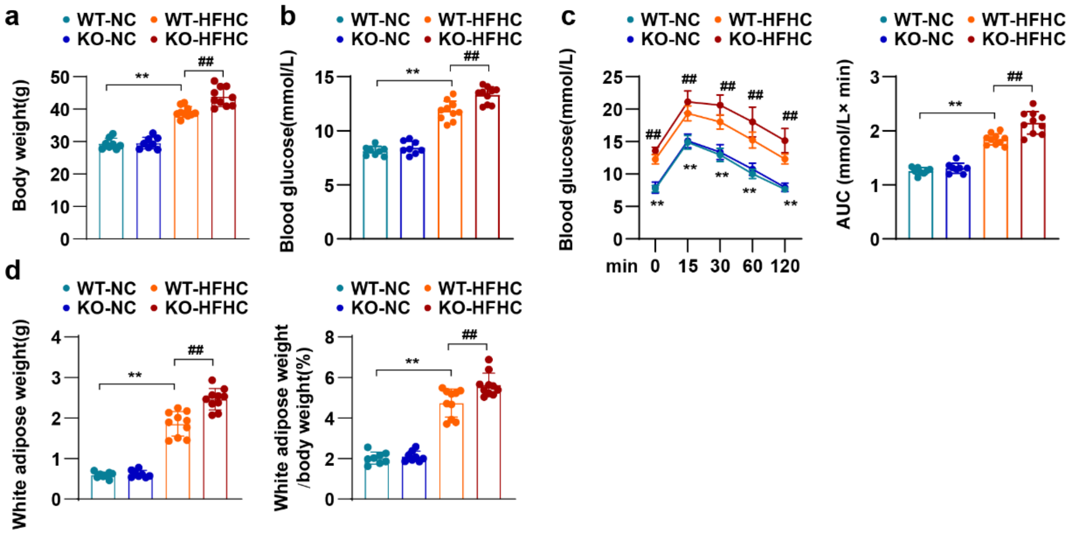

Supplementary Fig. 3: FAIM2 deletion exacerbates HFHC-induced MASH.

(a) Absolute body weight of FAIM2-WT and FAIM2-KO mice that were fed NC or HFHC (n = 8/NC group, n=10/HFHC group). (**b**) Mouse absolute blood glucose level of the indicated groups (n = 8/NC group, n=10/HFHC group). (**c**) Mouse absolute blood glucose level of GTT and GTT AUC of the indicated groups (n = 8/NC group, n=10/HFHC group). (**d**) Mouse absolute white adipose weight and white adipose/ body weight percentage of the indicated groups (n = 8/NC group, n=10/HFHC group). $^{**}p < 0.01$; $^{\#\#}p < 0.01$. The data were expressed as the means ± standard deviations. Statistical analysis was

carried out via one-way ANOVA.

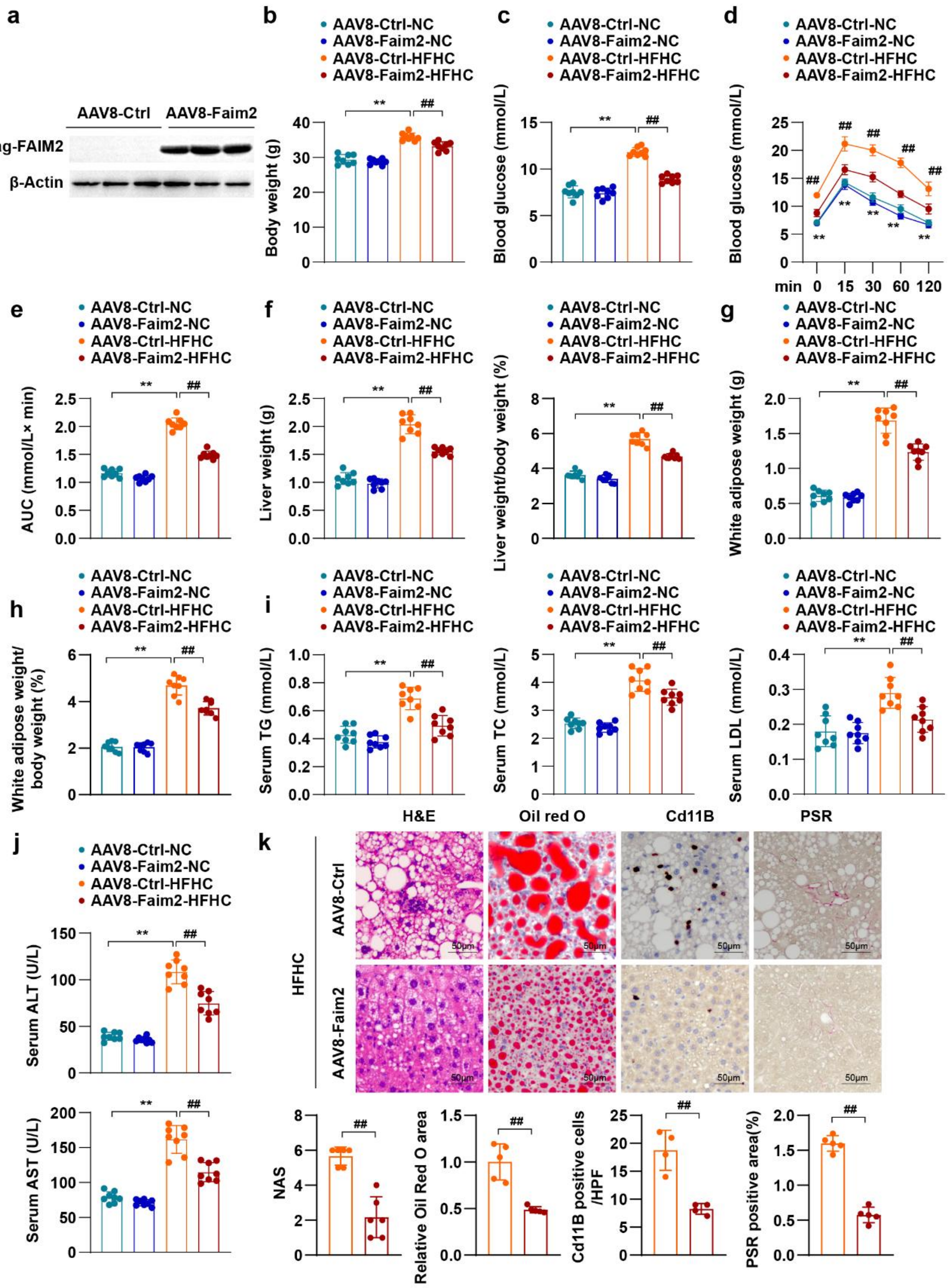

Supplementary Fig. 4: FAIM2 overexpression alleviates HFHC-induced MASH.

(a) Western blot images of FAIM2 protein expression in liver from AAV8-Faim2 and AAV8-control mice (n = 3). (**b)** Absolute body weight of AAV8-Ctrl and AAV8-Faim2 mice that were fed NC or HFHC (n = 8). (**c)** Mouse absolute blood glucose level of the indicated groups (n = 8). (**d and e)** Mouse absolute (d) blood glucose level of GTT and (e) GTT AUC of the indicated groups (n = 8). (**f)** Mouse absolute liver weight and liver weight/body weight percentage of the indicated groups (n = 8). (**g and h)** Absolute (g) white adipose weight and (h) white adipose/ body weight percentage of the indicated groups (n = 8). (**i and j)** Mouse absolute (i) serum TG, TC, LDL, (j) ALT and AST levels of the indicated groups (n = 8). (**k)** Images of H&E and Oil Red O; Cd11B IHC and PSR liver sections of the indicated groups (n = 5). Scale bars, 50 µm. High-power filed, HPF. $^{**}p < 0.01$; $^{\#\#}p < 0.01$. The data were expressed as the means ± standard deviations. Statistical analysis was carried out via one-way ANOVA or two-tailed Student's t test.

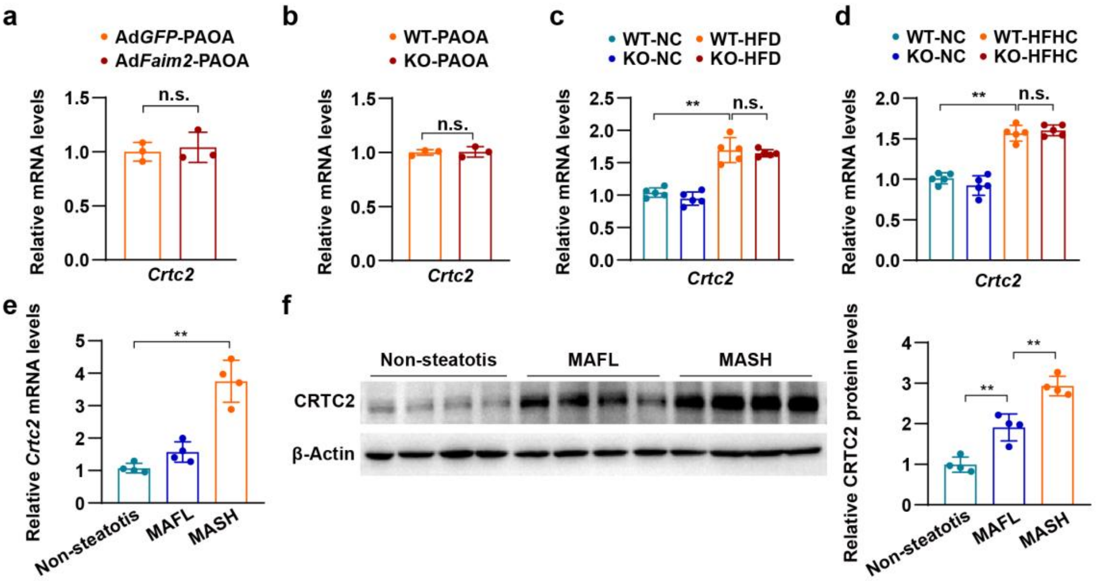

Supplementary Fig. 5: FAIM2 binds to CRTC2 and reduces its protein level.

(a and b) Normalized CRTC2 mRNA levels of wild-type hepatocytes, wild-type hepatocytes infected with Ad*GFP* or Ad*Faim2*, and FAIM2-KO hepatocytes (n = 3). **(c)** Normalized CRTC2 mRNA levels in the liver of NC- or HFD-fed FAIM2-KO and FAIM2-WT mice (n = 5). **(d)** Normalized CRTC2 mRNA levels of the indicated groups (n = 5). **(e and f)** Normalized CRTC2 mRNA and protein levels in the liver of non-steatotic donors and MAFLD individuals (n = 4). *$p < 0.05$, **$p < 0.01$; n.s., not significant. The data were expressed as the means ± standard deviations. Statistical analysis was carried out via one-way ANOVA or two-tailed Student's t test.

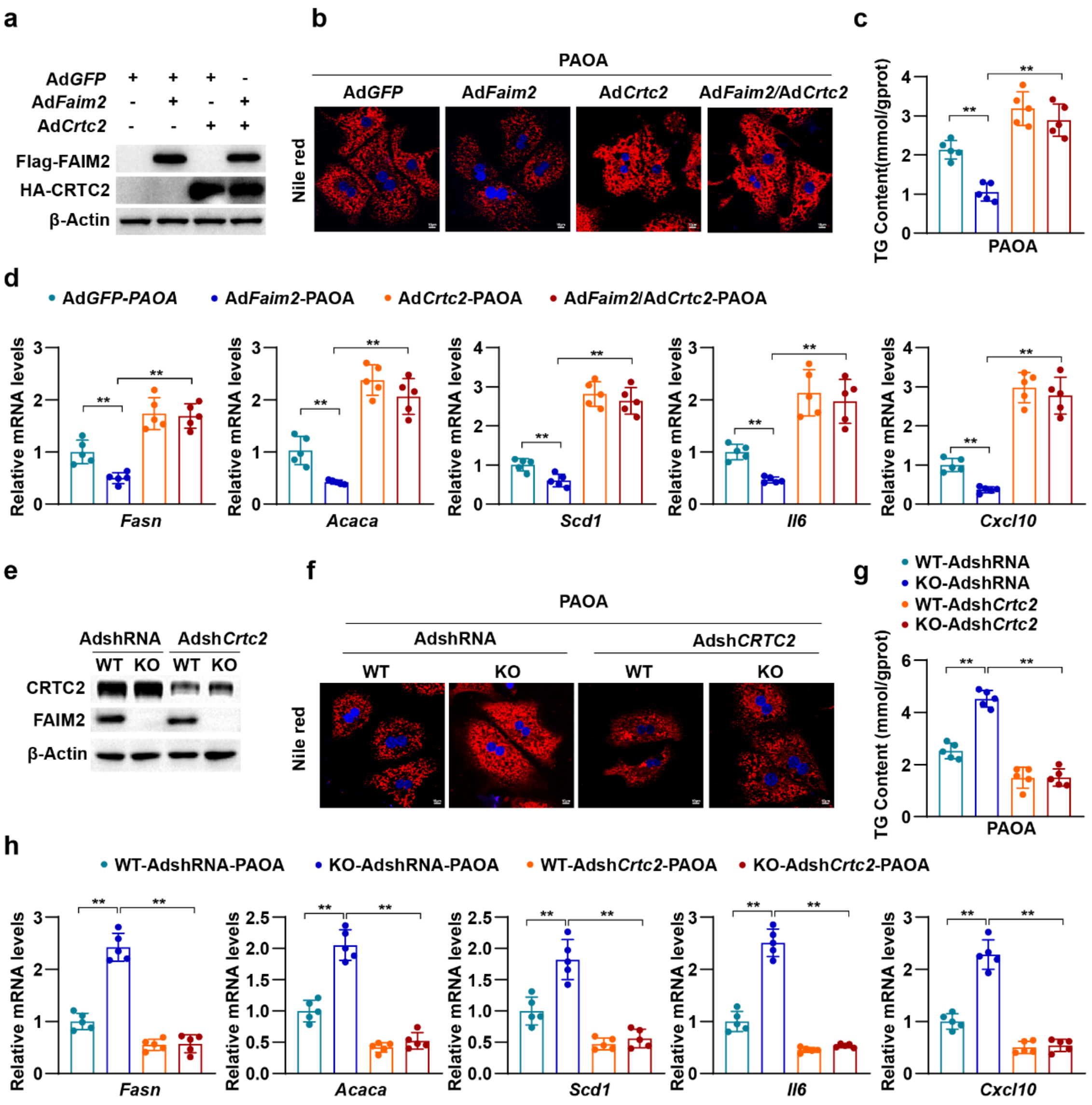

Supplementary Fig. 6: FAIM2 inhibits lipid synthesis via CRTC2.

(a) Western blot images of Flag-FAIM2 and HA-CRTC2 in primary hepatocytes infected with Ad*Faim2*, Ad*Crtc2* or their combination. **(b)** Nile Red staining images of the indicated groups (n = 3). Scale bar, 10 µm. **(c)** Absolute TG content of hepatocytes of the indicated groups (n = 5). **(d)** Normalized mRNA levels of the indicated groups (n = 5). **(e)** Western blot images of FAIM2 and CRTC2 in primary hepatocytes from FAIM2-KO or its control mice infected with Adsh*CRTC2* or AdshRNA. **(f)** Nile Red staining images of the indicated groups (n = 3). Scale bar, 10 µm. **(g)** Absolute TG content of hepatocytes of the

indicated groups (n = 5). **(h)** Normalized mRNA levels of the indicated groups (n = 5). $^{**}p < 0.01$. The data were expressed as the means ± standard deviations. Statistical analysis was carried out via one-way ANOVA.

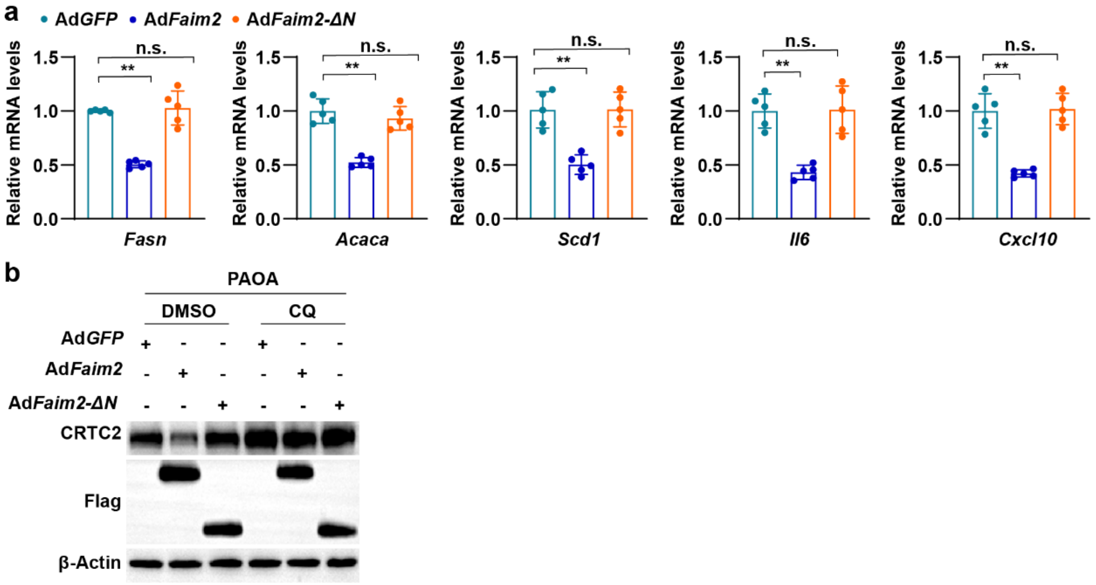

Supplementary Fig. 7: The N-terminal domain of FAIM2 is required for CRTC2 degradation.

(**a**) Normalized mRNA levels of wild-type hepatocytes infected with Ad*GFP*, Ad*Faim2* or Ad*Faim2-ΔN*. (ΔN, missing N-terminal domain). (n = 5). (**b**) Western blot images of indicated groups. $^{**}p < 0.01$; n.s., not significant. The data were expressed as the means ± standard deviations. Statistical analysis was carried out via one-way ANOVA.

Supplementary tables

Supplementary Table1: Demographic data and histological scores of human liver samples.

ID	Age	Sex	BMI	Steatosis	Lobular inflammation	Ballooning	NAS	Fibrosis stage
Patient 1	32	Male	27.9	0	0	0	0	F0
Patient 2	35	Male	34.2	0	0	0	0	F0
Patient 3	34	Female	30.04	0	0	0	0	F0
Patient 4	27	Female	31.61	0	0	0	0	F0
Patient 5	20	Female	30.46	0	0	0	0	F0
Patient 6	41	Female	31.1	2	0	0	2	F0
Patient 7	29	Female	34.83	2	0	0	2	F0
Patient 8	47	Male	45.69	2	0	0	2	F0
Patient 9	29	Female	34.9	2	0	0	2	F0
Patient 10	46	Female	29.2	1	0	0	1	F0
Patient 11	31	Male	39.64	2	1	2	5	F0
Patient 12	27	Male	40.3	2	1	2	5	F0
Patient 13	21	Male	46.7	3	2	2	7	F1
Patient 14	25	Female	42.44	3	1	2	6	F1
Patient 15	21	Female	44.6	3	2	1	6	F1

Supplementary Table 2: Primer sequence for construction of plasmids and adenovirus.

Name	Primers (5'-3')
Flag-FAIM2	F: TCGGGTTTAAACGGATCCGCCACCATGACCCAGGGAAAGCTCTCC
	R: GGGCCCTCTAGACTCGAGTTCTCGGTTAGTGCCAAAAAGCTGC
Flag-m-FAIM2	F: TCGGGTTTAAACGGATCCATGACCCAGGGAAAGCTCTCT
	R: GGGCCCTCTAGACTCGAGTTCCCGGTTGGTGCCAAAAAGC
GST-HA- FAIM2	F: TCGGGTTTAAACGGATCCGCCACCATGACCCAGGGAAAGCTCTCC
	R: GGGCCCTCTAGACTCGAGTTCTCGGTTAGTGCCAAAAAGCTGC
Flag-FAIM2(1-311)	F: TCGGGTTTAAACGGATCCATGACCCAGGGAAAGCT
	R: GGGCCCTCTAGACTCGAGTCAAAAAAGCTGCAGGAAGAAGG
Flag-FAIM2(101-311)	F: TCGGGTTTAAACGGATCCATGGTCTTTGTCAGAAAGGTC
	R: GGGCCCTCTAGACTCGAGTCAAAAAAGCTGCAGGAAGAAG
Flag-FAIM2(101-316)	F: TCGGGTTTAAACGGATCCATGGTCTTTGTCAGAAAGGTCTACACC
	R: GGGCCCTCTAGACTCGAGTCATTCTCGGTTAGTGCCAAAAAG
Flag-FAIM2(ΔLIR)	F: GGTGCCTCTCCACCCTAGCGACCCCAGCAGCAGCTCC
	R: GCTAGGGTGGAGAGGCACC
Flag-CRTC2	F: TCGGGTTTAAACGGATCCATGGCGACGTCGGGGG
	R: GGGCCCTCTAGACTCGAGTCATTGGAGCCGGTCACT
HA-CRTC2	F: TCGGGTTTAAACGGATCCATGGCGACGTCGGGGG
	R: GGGCCCTCTAGACTCGAGTCATTGGAGCCGGTCACT
GST-HA-CRTC2	F: TCGGGTTTAAACGGATCCATGGCGACGTCGGGGG
	R: GGGCCCTCTAGACTCGAGTCATTGGAGCCGGTCACT
HA-CRTC2(1-144)	F: TCGGGTTTAAACGGATCCGCCACCATGGCGACGTCGGGGGC
	R: GGGCCCTCTAGACTCGAGCTATCGCCAGCTAGACTCTGGG
HA-CRTC2(136-320)	F: TCGGGTTTAAACGGATCCGCCACCATGTCTCCTCCCCCAGAGTCTAGCT
	R: GGGCCCTCTAGACTCGAGCTAGCTGATGCCCAGGTGAG
HA-CRTC2(316-693)	F: TCGGGTTTAAACGGATCCGCCACCATGCACCTGGGCATCAGC
	R: GGGCCCTCTAGACTCGAGTCATTGGAGCCGGTCACTGCG
Flag-m-FAIM2(ΔLIR)	F: CTGTGCCACTCCATCCAAGCGACCCCAGCGGCAGC
	R: GCTTGGATGGAGTGGCACAG
Ad-m-FAIM2	F: GCTAGCGATATCGGATCCGCCACCATGACCCAGGGAAAGCTCTCTG
	R: GTCCTTGTAATCACTAGTTTCCCGGTTGGTGCCAAAAAG
Ad-m-NEDD4L	F: GGCTAGCGATATCGGATCCATGGCGACCGGGCTTGGG
	R:

	AGCGTAATCTGGAACATCGTATGGGTAACTAGTATCCACACCTTCGA AGCCTTG
Ad-m-CRTC2	F: TCGGGTTTAAACGGATCCATGGCGACGTCAGGGGCG
	R: GGGCCCTCTAGACTCGAGTCACTGTAGCCGATCACTACGGAATGAG TCCT
Ad-m-shCRTC2	Sh: GAGGACTCATTCCGTAGTGAT
Ad-m-FAIM2(ΔLIR)	F: TCGGGTTTAAACGGATCCATGACCCAGGGAAAGCTCTCT
	R: GGGCCCTCTAGACTCGAGTTCCCGGTTGGTGCCAAAAAGC

Supplementary Table 3: Primer sequences for RT-PCR detection.

Gene name	Forward primer (5'-3')	Reverse primer (5'-3')
FAIM2 - human	ATGGCCTACCTCACTGGGAT	TGGAAGCTGAAGACGGTGAC
Faim2 - mouse	GGCTGTGGTGGCTCTCTTTA	GGGAAATGTCTCCTGGGTCC
Cd36 - mouse	GACTGGGACCATTGGTGATGA	AAGGCCATCTCTACCATGCC
Fasn - mouse	TGGGTTCTAGCCAGCAGAGT	ACCACCAGAGACCGTTATGC
Scd1 - mouse	TCTTCCTTATCATTGCCAACACCA	GCGTTGAGCACCAGAGTGTATCG
Pparg - mouse	ATTCTGGCCCACCAACTTCGG	TGGAAGCCTGATGCTTTATCCCCA
Acaca - mouse	GGCCAGTGCTATGCTGAGAT	AGGGTCAAGTGCTGCTCCA
Ccl2 - mouse	TACAAGAGGATCACCAGCAGC	ACCTTAGGGCAGATGCAGTT
Cxcl10 - mouse	ATGACGGGCCAGTGAGAATG	ATGATCTCAACACGTGGGCA
Col1a1 - mouse	TGCTAACGTGGTTCGTGACCGT	ACATCTTGAGGTCGCGGCATGT
Col4a1 - mouse	AACAACGTCTGCAACTTCGC	CTTCACAAACCGCACACCTG
Il6- mouse	TAGTCCTTCCTACCCCAATTTCC	TTGGTCCTTAGCCACTCCTTC
Crtc2 - mouse	TCTCAGGCCTGCTTAGTGC	TTGAATTGCTCCAGATTCCCCA
Actb - mouse	GTGACGTTGACATCCGTAAAGA	GCCGGACTCATCGTACTCC
ACTB- human	CATGTACGTTGCTATCCAGGC	CTCCTTAATGTCACGCACGAT

Supplementary Table 4: Antibody for western blotting.

Anbibody	Manufacturer	Catalogue number	Source of species	Dilution
FAIM2	Santa Cruz	sc-398737	Mouse	1:1000
CRTC2	Proteintech	12497-1-AP	Rabbit	1:1000
LAMP1	CST	15665	Mouse	1:1000
Ubiquitin	CST	43124	Rabbit	1:1000
LC3	ABclonal	A5618	Rabbit	1:1000
P62	ABclonal	A19700	Rabbit	1:1000
LC3B	CST	83506	Rabbit	1:1000
Flag	ABclonal	AE092	Rabbit	1:200
HA	ABclonal	AE105	Rabbit	1:200
Flag	MBL	M185-3L	Mouse	1:1000
HA	MBL	M180-3	Mouse	1:1000
β-Actin	ABclonal	AC026	Mouse	1:5000
