# Supplementary figures and images for: Fas apoptotic inhibitor molecule 2 mitigates metabolic dysfunction-associated fatty liver disease through autophagic CRTC2 degradation
Source: Exp Mol Med. 2025 Oct 7;57(10):2331–43. doi: 10.1038/s12276-025-01559-1 (PMC12586438; doi:10.1038/s12276-025-01559-1)

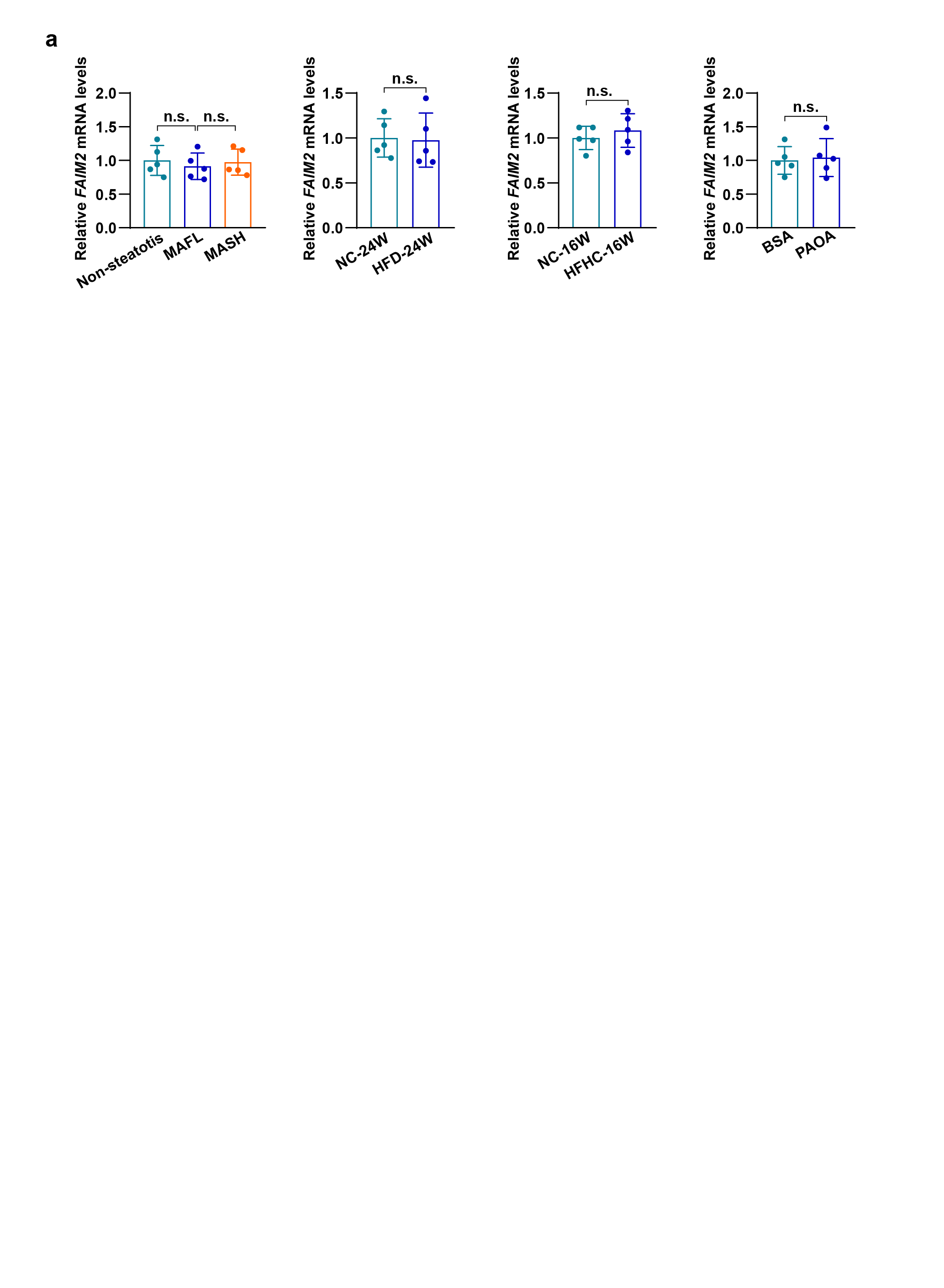

Supplement: Supplementary file 2 — Supplementary Fig. 1 [file 12276_2025_1559_MOESM2_ESM.png]

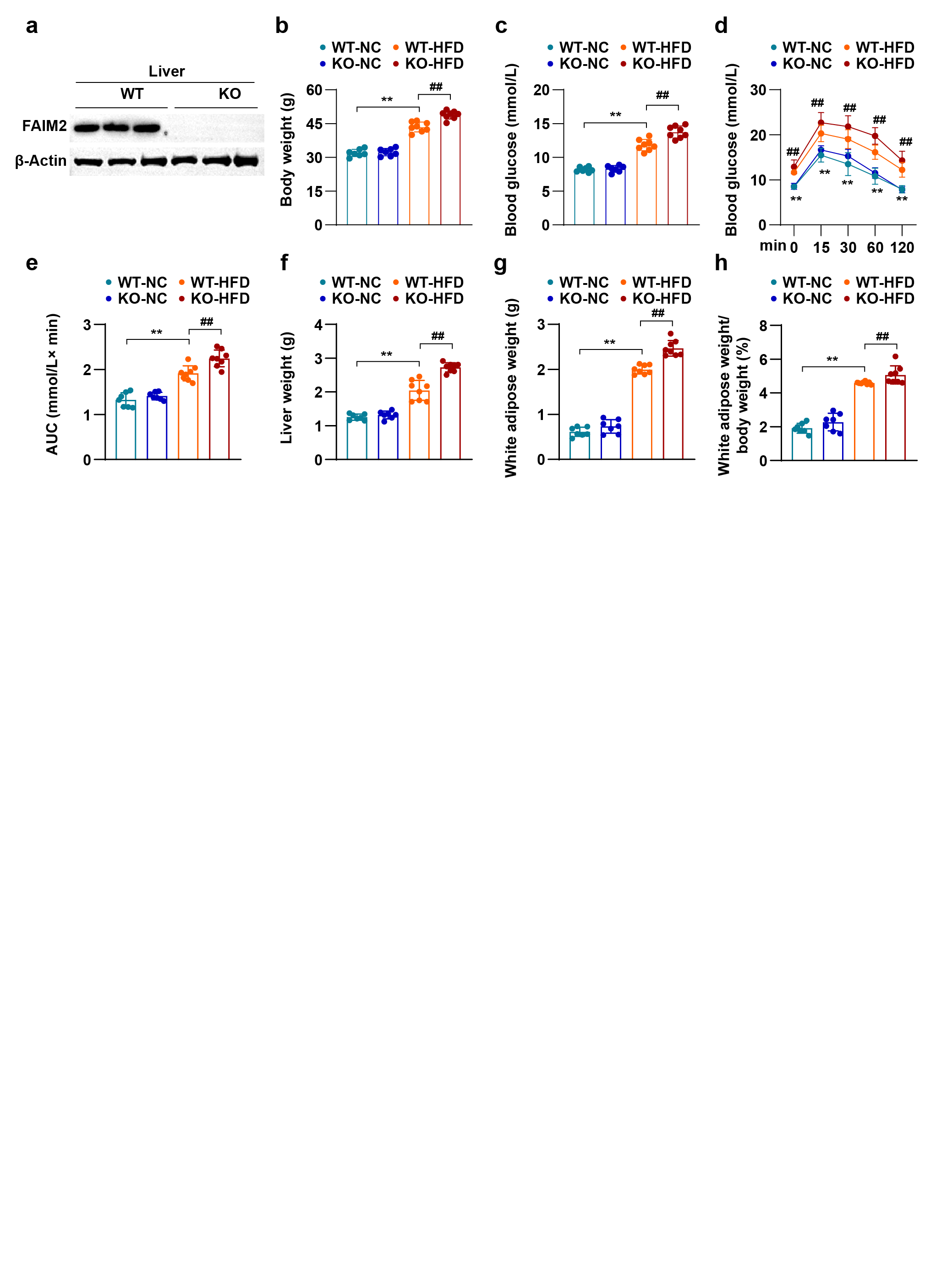

Supplement: Supplementary file 3 — Supplementary Fig. 2 [file 12276_2025_1559_MOESM3_ESM.png]

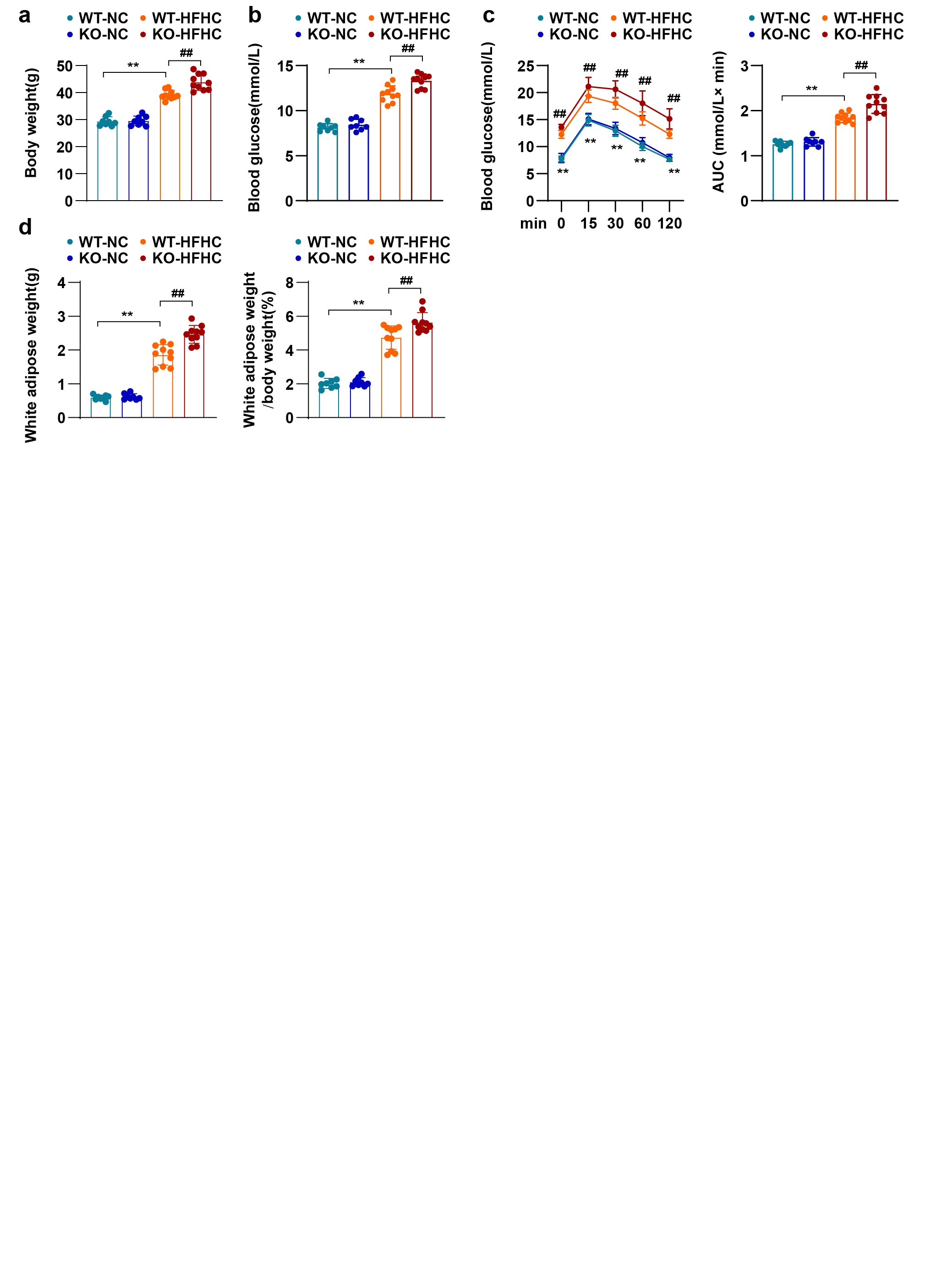

Supplement: Supplementary file 4 — Supplementary Fig. 3 [file 12276_2025_1559_MOESM4_ESM.png]

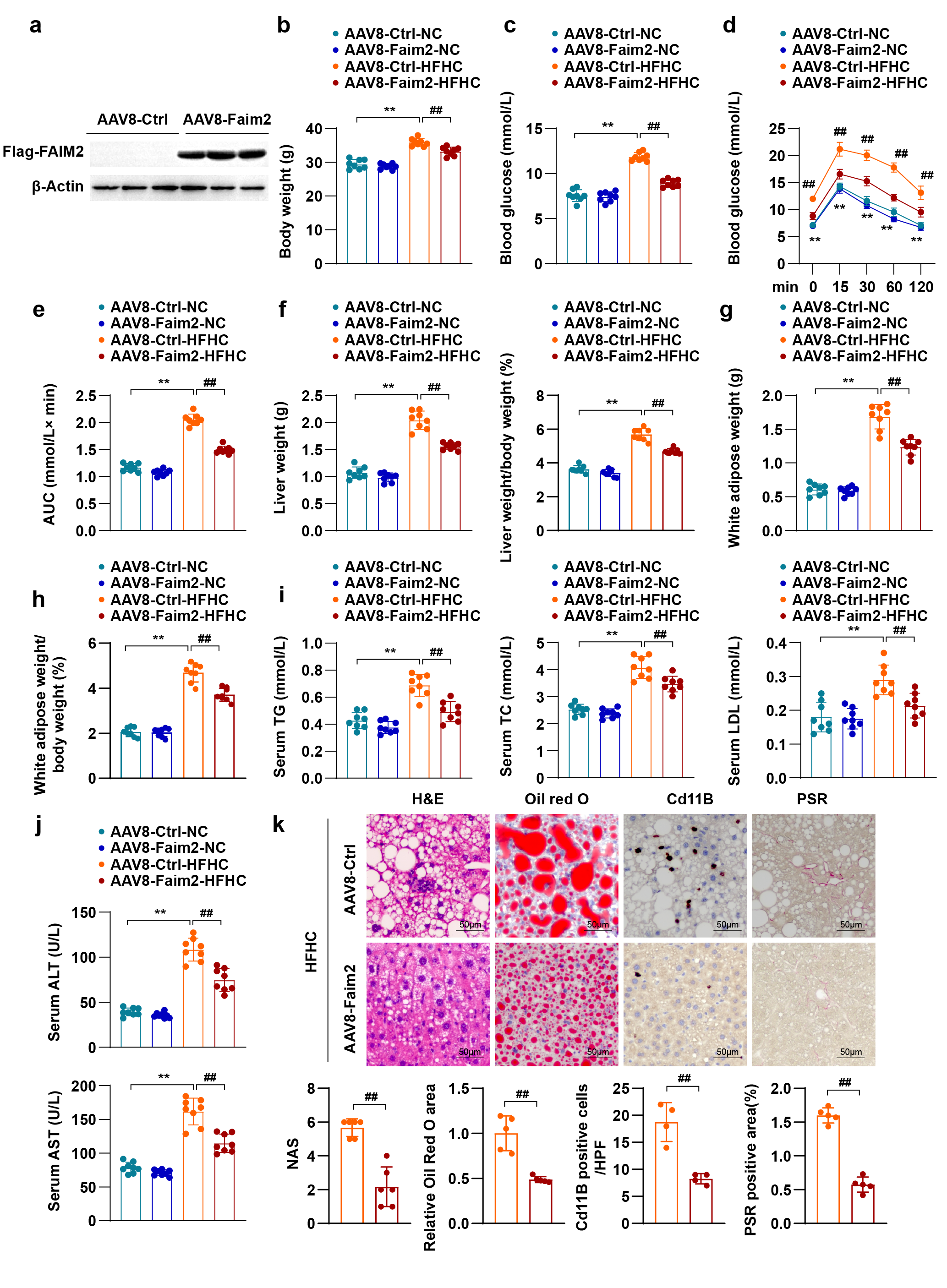

Supplement: Supplementary file 5 — Supplementary Fig. 4 [file 12276_2025_1559_MOESM5_ESM.png]

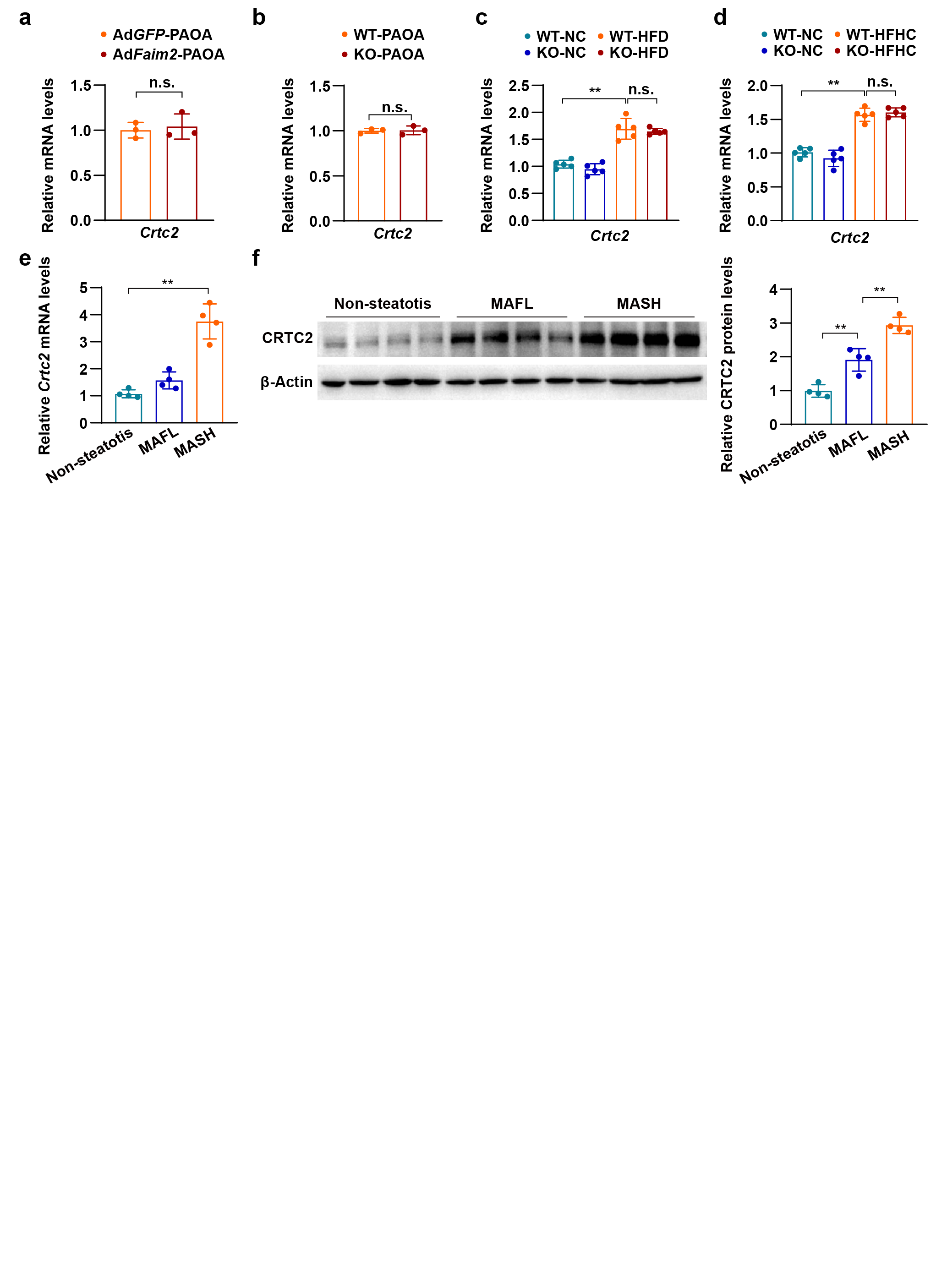

Supplement: Supplementary file 6 — Supplementary Fig. 5 [file 12276_2025_1559_MOESM6_ESM.png]

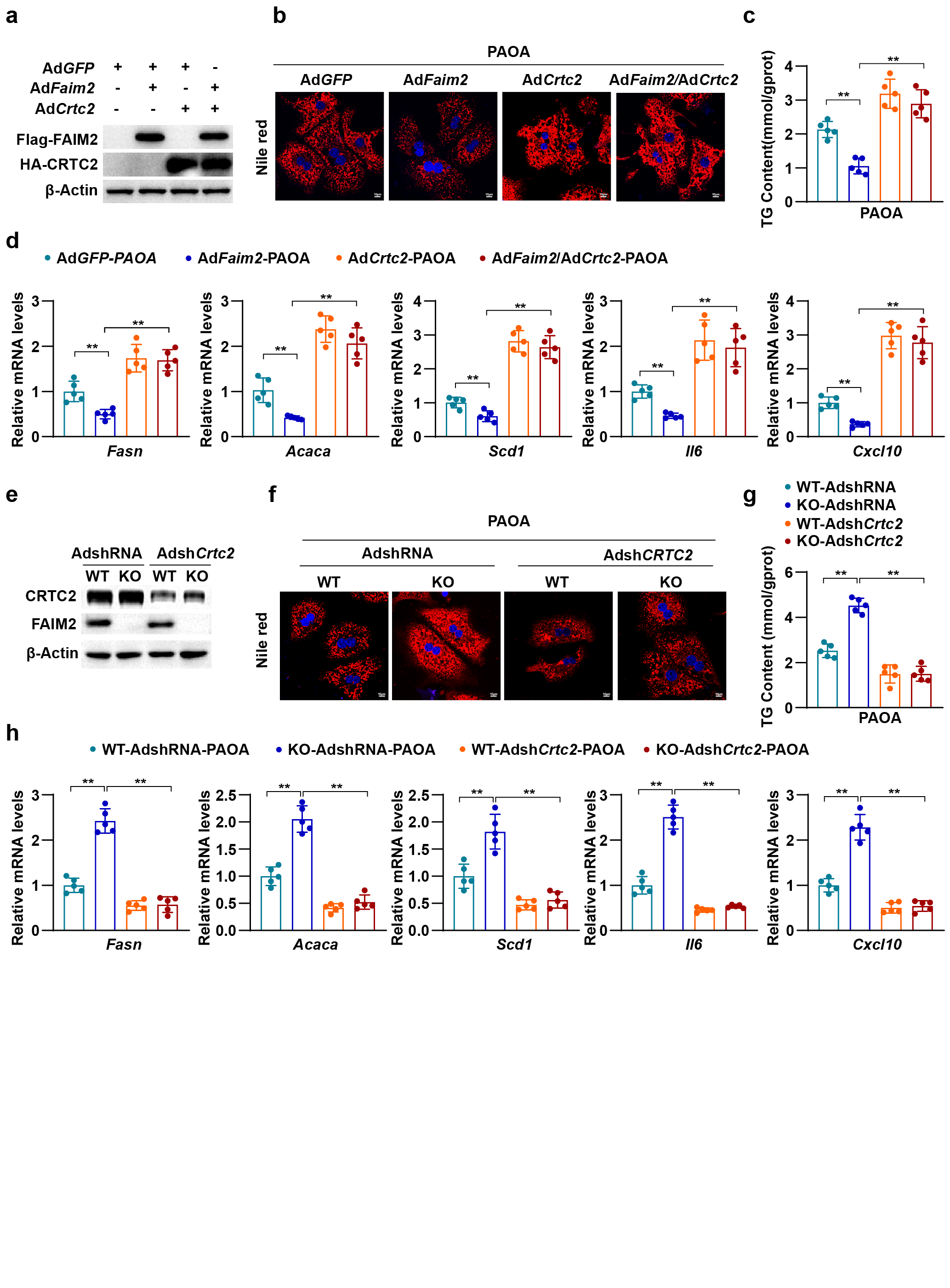

Supplement: Supplementary file 7 — Supplementary Fig. 6 [file 12276_2025_1559_MOESM7_ESM.png]

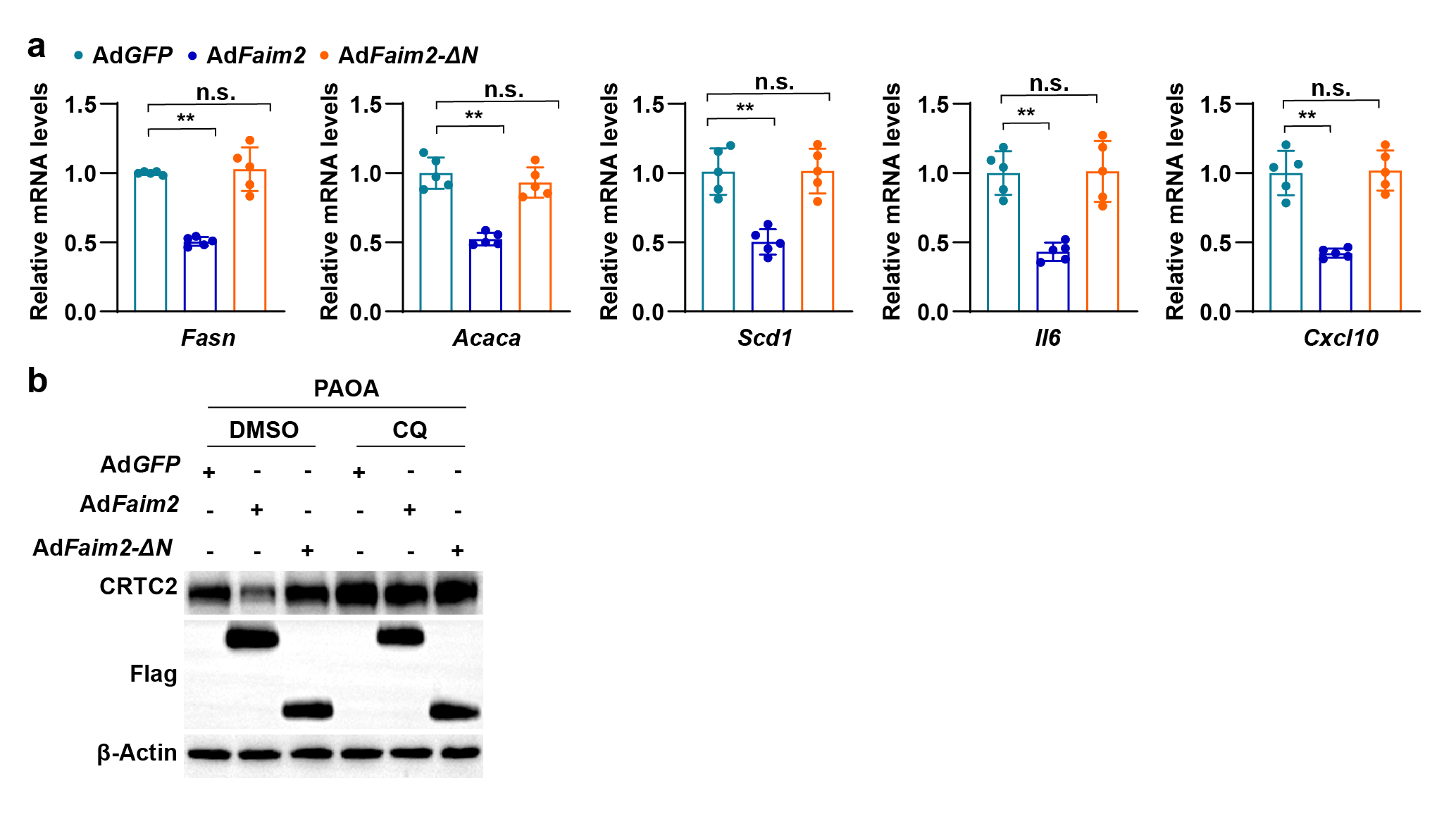

Supplement: Supplementary file 8 — Supplementary Fig. 7 [file 12276_2025_1559_MOESM8_ESM.png]
